# Potential of medicinal plants as antimalarial agents: a review of work done at Kenya Medical Research Institute

**DOI:** 10.3389/fphar.2023.1268924

**Published:** 2023-10-20

**Authors:** Beatrice Irungu, Erick Okari, Mary Nyangi, Sospeter Njeru, Lilian Koech

**Affiliations:** ^1^ Center for Traditional Medicine and Drug Research, Kenya Medical Research Institute, Nairobi, Kenya; ^2^ Physcial Sciences Department, South Eastern Kenya University, Kitui, Kenya

**Keywords:** malaria, medicinal plants, antimalarial, antiplasmodial, *Plasmodium falciparum*, drug discovery, cytotoxicity

## Abstract

**Background:** Medicinal plants have traditionally been used as remedies against malaria. The present review attempted to compile data on scientific research evidence on antimalarial medicinal plants screened at Kenya Medical Research Institute (KEMRI), Center for Traditional Medicine and Drug (CTMDR) Research from January 2003 to December 2021.

**Methods:** A systematic review was conducted using a predefined protocol based on PRISMA. Search was performed in Google Scholar and PubMed. One hundred and eight journal articles were identified 37 of which published on antimalarial/antiplasmodial work. Thirty journal articles with at least one author from KEMRI-CTMDR and accessible in full were selected for analysis. Relevant data was captured in MS Excel format and descriptive statistics, percentages and tables used to summarize the findings.

**Results:** Assessment of individual plant species was considered as an independent study resulting in 1170 antiplasmodial/antimalarial tests done from 197 plant species. One hundred and fifty plant species were screened *in vitro*, one *in vivo* and 46 were both *in vivo* and *in vitro.* Three hundred and forty-four of tests reported good activity (IC_50_ < 10 μg/mL or parasite suppression rate of ≥50%), 414 moderate activity (IC_50_ values of 10–49 μg/mL or parasite suppression rate of 30%–49%) and 412 were reports of inactivity (IC_50_ ˃ 50 μg/mL or parasite suppression rate of <30%). *Fuerstia africana* and *Ludwigia erecta* were reported to have the highest activities, with IC_50_ < 1 μg/mL against *Plasmodium falciparum* D6 strain and chemosuppression in mice at an oral dose of 100 mg/kg, was reported as 61.9% and 65.3% respectively. Fifty five antimalarial/antiplasmodial active compounds isolated from eight plant species were reported with resinone (**39**) having the best activity (IC_50_ < 1 μg/mL).

**Conclusion:** Though 344 of tests reported promising antimalarial activity, it was noted that there was limited evaluation of these plants in animal models, with only 9.0% (105/1170) studies and no clinical trials. This highlights an important research gap emphasizing the need for drug development studies that aim to progress study findings from preclinical to clinical studies. There is still need for extensive research on promising plant species aimed at developing new plant based antimalarial drugs.

## 1 Introduction

Morbidity and mortality caused by malaria is still a public health concern despite the fact that it is a curable and preventable disease. World Malaria Report of 2022 reveals that between 2019 and 2020, estimated malaria cases increased from 218 to 232 million, and deaths from 544 000 to 599 000 in the World Health Organization (WHO) African Region WHO (2022). In Kenya, malaria remains a major public health problem accounting for an estimated 13%–15% of outpatient consultations. The *Plasmodium falciparum* parasite, which causes the most severe form of the disease, accounts for more than 99% of infections. Malaria prevalence in Kenya varies considerably by season and across different geographic zones. This is because transmission and infection risks are mainly determined by altitude, rainfall patterns and temperature (Division of National Malaria Programme DNMP [Kenya] and International Classification of Functioning, Disability, and Health ICF, 2021). Key malaria control and prevention strategies that have been employed in Kenya include use of insecticide treated nets, intermittent preventive treatment during pregnancy (IPTp) using sulfadoxine pyrimethamine, indoor residual spraying and adoption of artemisinin combination therapy (ACT). However, the adaptation of the mosquitoes to insecticides and emergence and spread of drug resistant parasites, especially *P*. *falciparum*, is a drawback to these interventions. The WHO (2022) confirmed emergency of partial resistance to artemisinin drugs in some African countries, namely,: Rwanda, Eritrea, and Uganda. The possibility of the spread of artemisinin resistant parasites to other malaria endemic regions in Africa is inevitable. Therefore, the challenge to eliminate malaria remains significant hence the need for new agents that are cheap, safe, readily available, active against sensitive and drug resistant *Plasmodium* parasites or act in combination with existing drugs.

Medicinal plants have played a major role in discovery and development of antimalarial drugs. It is expected that medicinal plants would still serve as a source of new drug leads given their chemodiversity (Batista et al., 2009). Several studies have documented medicinal plants used in management of malaria by various local communities in Kenya (Muthaura et al., 2007a; Njoroge and Bussmann, 2007; Gathirwa et al., 2011; Mukungu et al., 2016). Continued research on Kenyan medicinal plants has offered plants extracts and purified secondary metabolites with potent antimalarialantiplasmodial activities (Muiva et al., 2009; Irungu et al., 2014; 2015; Muthaura et al., 2015b).

In this review we summarize research evidence on toxicity, cytotoxicity, antimalarial and antiplasmodial properties of medicinal plant extracts and secondary metabolites evaluated at Kenya Medical Research Institute, Center for Traditional Medicine and Drug Research (KEMRI-CTMDR) between January 2003 and December 2021. This review covers a period within which there was increased research activities on screening medicinal plants for antimalarial properties providing a recent outlook of our drug discovery efforts. We acknowledge that other Kenyan institution have documented medicinal plants with antimalarial activity. However, this review chose to exclusively focus on work done at KEMRI due to its renowned expertise in human health research, including rationalization of traditional medicine in Kenya.

## 2 Methods

### 2.1 Systematic review

A systematic review was conducted using a predefined protocol based on Preferred Reporting Items for Systematic Reviews and Meta-Analyses (PRISMA) (Page et al., 2021) guidelines including; literature search to identify potential articles, assessing the relevance of the articles quality and data extraction. The search was performed in Google Scholar and PubMed covering the period January 2003 to December 2021 and was limited to original English journal articles whose full text were accessible. Literature search was performed using key terms such as: Kenyan medicinal plants with antiplasmodial/antimalarial activities, antimalarial studies at Kenya Medical Research Institute (KEMRI), Center for Traditional Medicine and Drug Research (CTMDR), Kenyan antimalarial herbal remedies. We also searched with individual names of past and present Research Scientists working at KEMRI-CTMDR.

### 2.2 Inclusion and exclusion criteria

**FIGURE 1:**
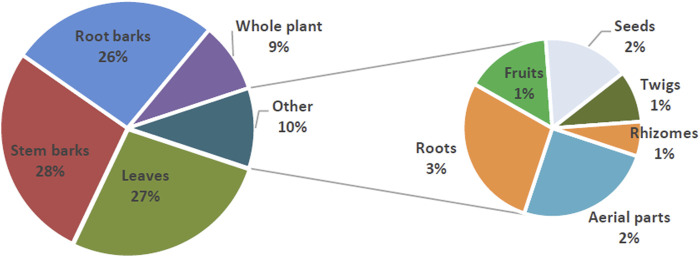
Frequency distribution of plant parts used to prepare extracts.

After a web search on pharmacological activities of medicinal plants screened at KEMRI-CTMDR, 108 journal articles were identified, 37 of which reported on antimalarial/antiplasmodial activities. Seven articles, did not meet our inclusion criteria since they either did not have an author from CTMDR, reported on synthetic compounds, were non-open access or were partially accessed (abstract only) as shown in Figure 1. Thirty journal articles that met our selection criteria were selected for analysis.

### 2.3 Data screening and extraction

The articles were further analyzed based on the originality of reported data determined by the description of the study design. Data was captured in excel format and the following information from each eligible journal article was extracted; article title, plant botanical name, family, plant collection site, part(s) of the plant used, type of study (*in vitro* or *in vivo*), *Plasmodium* strain tested, IC_50_ values,% chemosuppression, isolated compound (s), cytotoxicity (CC_50_ or IC_50_), toxicity (LD_50_) and extraction solvent used. Descriptive statistics was used to summarize the findings.

## 3 Results

### 3.1 Medicinal plants screened for antimalarial/antiplasmodial activity

KEMRI-CTMDR Research Scientists have published data on 197 plant species and their potential antimalarial/antiplasmodial activities within a period of 18 years (January 2003 to December 2021). An extensive search and abstract screening within this period revealed 30 articles with at least one researcher from KEMRI-CTMDR as the main/co-author. Out of the 30 journal articles considered in this review, 66.7% (20/30) of the studies were done by local collaborators compared to international collaborators at 33.3% (10/30). The most preferred journal was *Journal of Ethnopharmacology* with 11 publications out of the 30 articles analyzed (Table 1). Twenty six out of the 30 articles, focused on plants that were collected from within Kenya while four articles investigated plant materials that were collected from outside Kenya (Table 1).

**TABLE 1:** Site of plant collection, nature of collaboration and journal published.

Collection site	Nature of collaboration	Journal published	References
Meru, Kenya	International	*Fitoterapia*	Rukunga et al. (2008)
Meru, Kenya	Local	*Journal of Ethnopharmacology*	Gathirwa et al. (2008)
Meru, Kenya	local	*Journal of Ethnopharmacology*	Muthaura et al. (2015b)
Meru, Kenya	local	*Journal of Natural Medicines*	Gathirwa et al. (2007)
Kilifi, Kenya	Local	*Journal of Ethnopharmacology*	Rukunga et al. (2009)
Kilifi, Kenya	International	*Journal of Ethnopharmacology*	Gathirwa et al. (2011)
Kajiado, Embu, Baringo; Kenya	Local	*African Journal of Pharmacology and Therapeutics*	Rotich et al. (2015)
Kajiado, Kenya	International	*Natural Product Research*	Muiva-Mutisya et al. (2017)
Kajiado, Kenya	Local	*South African Journal of Botany*	Kigondu et al. (2011)
Mombasa, Kenya	Local	*Journal of Pathogens*	Udu et al. (2021)
Meru and Mombasa; Kenya	Local	*South African Journal of Botany*	Irungu et al. (2007)
Kisumu, Kenya	Local	*Journal of Ethnopharmacology*	Orwa et al. (2013)
Kwale, Kenya	Local	*African Journal of Health Sciences*	Nyangacha et al. (2012)
Meru, Kenya	Local	*Phytotherapy Research*	Muthaura et al. (2007b)
Kwale, Kenya	Local	*Journal of Ethnopharmacology*	Muthaura et al. (2015b)
Central Kenya	Local	*Journal of Ethnopharmacology*	Kigondu et al. (2009)
West Pokot, Kenya	Local	*European Journal of Medicinal Plants*	Wachira et al. (2018)
Machakos, Kenya	International	*African Journal of Traditional, Complementary and Alternative Medicines*	Mutai et al. (2008)
Makueni, Kenya	International	*Phytochemistry Letters*	Muiva et al. (2009)
Nandi, Kenya	Local	*African Journal of Pharmacology and Therapeutics*	Jeruto et al. (2015)
Nairobi, Kenya	International	*Journal of Ethnopharmacology*	Irungu et al. (2015)
Kiambu, Kenya	International	*Molecules*	(Irungu et al., 2014)
Arusha, Tanzania	International	*Journal of Medicinal Plants Research*	(Ng’etich et al., 2020))
Arusha, Tanzania	Local	*African Journal of Pharmacology and Therapeutics*	Kangethe et al. (2016)
Baka Pygmies of the Dja Biosphere Reserve in Cameroon	International	*Journal of Natural Products*	Fotie et al. (2006)
Uganda	Local	*Journal of Ethnopharmacology*	Obbo et al. (2019)
Meru and Kilifi, Kenya	Local	*Journal of Ethnopharmacology*	Kirira et al. (2006)
OAU campus, Ile-Ife, Nigeria	International	*Journal of Herbs, Spices and Medicinal Plants*	Adebajo et al. (2013)
Ngong’ forest, Kajiado County in Kenya	Local	*African Journal of Pharmacology and Therapeutics*	Irungu et al. (2012)
Nairobi, Kenya	International	*Acta Tropica*	Yenesew et al. (2012)

In this paper, each assessment of a plant species was treated as a separate study, which means that depending on the number of plant species examined, an article could encompass multiple studies. In total there were 1170 antiplasmodial/antimalarial tests done from 197 plant species. One hundred and fifty (76.1%) plant species were screened *in vitro*, one (0.5%) *in vivo* and 46 (23.4%) were both *in vivo* and *in vitro*. Majority of studies reported crude extracts except three, where fraction blends obtained separately from *Gongronema latifolium* (Benth.) K. Schum*, Artemisia annua* L. and *Lippia kituiensis* Vatke were evaluated (Adebajo et al., 2013; Kangethe et al., 2016; Ng’etich et al., 2020).

### 3.2 Diversity of plants evaluated

**FIGURE 2:**
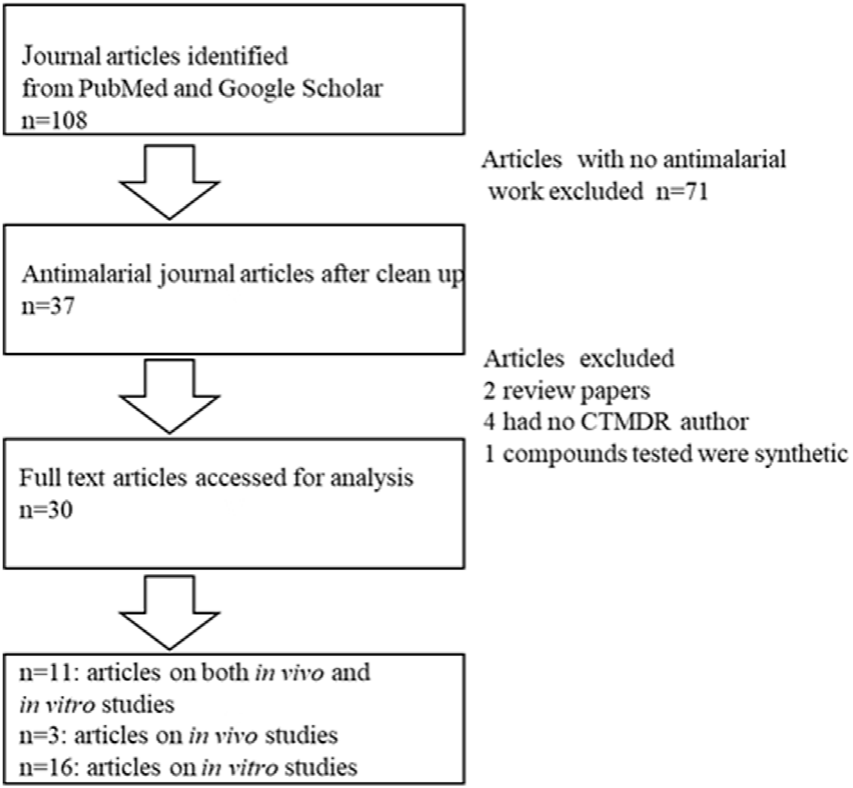
Flow chart describing journal articles selection strategy.

Of the 197 plants species, the most studied plant families were Asteraceae 16 (8.1%), Verbenaceae, 9 (4.6%), Rubiaceae*,* 8 (4%), Fabaceae*,* 7 (3.6%) and Leguminosae*,* 7 (3.6%). The most investigated plant species were; *Rotheca myricoides* (Hochst.) Steane and Mabb *Azadirachta indica* A. Juss., *Rhus natalensis* Bernh. ex Krauss, *Turraea robusta* (Hochst.) Benth., *Ximenia americana* L.*, Vernonia auriculifera* Hiern, *Toddalia asiatica* (L.) Lam.*, Maytenus undata* (Thunb.) Blakelock, *Lannea schweinfurthii* (Engl.) Engl., *Zanthoxylum chalybeum* Engl.*, Harrisonia abyssinica* Oliv. *Fuerstia africana* Oliv. and *Asparagus racemosus* Willd. Leaves, 85 (27%), stem barks, 87 (28%), root barks, 83 (26%) and whole plant 28 (9%) were the most common parts of the plants used to prepare extracts (Figure 2). Crude extracts dominated in the tests compared to tests done using isolated compounds at 1072 (91.6%) and 98 (8.4%), respectively. Moreover, a majority of the extracts were organic 401 (67.3%) compared to aqueous extracts 195 (32.7%). In ascending order: 1:1 mixture dichloromethane: methanol, 9 (1.5%), hexane, 13 (2.2%), chloroform, 13 (2.2%), petroleum ether, 17 (2.9%), ethyl acetate, 18 (3%), dichloromethane, 19 (3.2%), water, 195 (32.7%) and methanol 309 (51.8%) were the most frequent extraction solvents used.

### 3.3 *In vitro* and *in vivo* activities of plant extracts

The activities were divided into three categories; good (IC_50_ values <10 μg/mL or suppression rate of ≥50%), moderate (IC_50_ values of 10 μg/mL–49 μg/mL or suppression rate of 30%–49%) and inactive (IC_50_ values ˃ 50 μg/mL or suppression rate of <30%) (Waiganjo et al., 2020). In general, 344 (29.4%) of the antiplasmodial tests reported good activity, 414 (35.4%) moderate activity and 412 (35.2%) were reports of inactivity. For the *in vitro* tests (Supplementary Table S1), inactive reports were the majority 386 (33%) followed by moderate activity 379 (32.4%) and good activity 300 (25.6%). Of the 300 *in vitro* studies with good activity, 177 (59%) were active with IC_50_ between 5 and 10 μg/mL while 123 (41%) were highly active with IC_50_ < 5 μg/mL (Table 2). On the other hand, a majority of *in vivo* tests reported good activity 44 (41.9%) (Table 3) followed by moderate activity 35 (33.3%) and 26 (24.8%) reported inactivity (Supplementary Table S2). Plant species that were commonly reported to display promising antiplasmodial activities in different studies included; *T*. *robusta* which exhibited good antiplasmodial activity in 8 out of 13 tests (61.5%), *T*. *asiatica,* 14 out of 26 tests (53.8%), *Erythrina burttii* Baker f., 12 out of 22 tests (54.5%) and *M*. *undata,* 10 out of 14 tests (71.4%).

**TABLE 2:** Plant extracts with highest antiplasmodial activity (IC_50_ < 5 μg/ml).

Plant screened	Plant family	Part used	Solvent used	Parasite strain	IC_50_ ug/ml	Cytotoxicity/LD_50_	References
*Holarrhena floribunda*	Apocynaceae	Stem bark	Aqueous extract	W2	1.02	n.d	Fotie et al. (2006)
			Ethanoic extract	D6	4.33	n.d	
			Chloroform	W2	2.29	n.d	
*Harrisonia abyssinica*	Simaroubaceae	Stem barks	DCM	K1	4.4	n.d	Irungu et al. (2007)
*Vernonia lasiopus*	Asteraceae	Root barks	DCM	K1	4.7	>90 μg/mL	
				NF54	4.9		
*Warbugia ugandensis*	Canellaceae	Stem barks	DCM	K1	1.4	0.34 μg/mL	
				NF54	2.2		
*Maytenus undata*	Celastraceae	Leaves	Water	D6	0.95	n.d	Muthaura et al. (2015a)
				W2	1.9	n.d	
		Root barks	Methanol	W2	4.9	n.d	
			Methanol	D6	4.4	n.d	
*Maytenus senegalensis*	Celastraceae	Root barks	Methanol	D6	4.7	n.d	
*Tabernaemontana pachysiphon*	Apocynaceae	Fruits	Water	D6	4.8	n.d	
				W2	3.4	n.d	
			Methanol	D6	3.9	n.d	
*Vernonia amygdalina*	Asteraceae	Leaves	Water	W2	3.8	n.d	
			Methanol	D6	4.9	n.d	
*Warburgia stuhlmannii*	Canellaceae	Stem barks	Methanol	D6	1.8	n.d	
				W2	2.3	n.d	
*Zehneria scabra*	Cucurbitaceae	Whole plant	Methanol	W2	1.8	n.d	
*Ziziphus mucronata*	Rhamnaceae		methanol	D6	4.4	n.d	
*Zanthoxylum chalybeum*	Rutaceae	Root barks	Water	W2	3.1	n.d	
			Methanol	D6	3.7	n.d	
				W2	2.9	n.d	
*Zanthoxylum chalybeum*	Rutaceae	Root barks	Water (K)	ENT30	2.32	n.d	Rukunga et al. (2009)
			Methanol (K)	ENT30	3.14	n.d	
			Water (T)	NF54	3.65	n.d	
				ENT30	2.88	n.d	
*Cyperus articulatus*	Cyperaceae	Rhizomes	Methanol	NF54	4.84	n.d	
*Erythrina burtii*	Fabaceae	Root barks	Acetone	D6	0.97	n.d	Yenesew et al. (2012)
				W2	1.73	n.d	
		stem barks		D6	2.6	n.d	
				W2	2.9	n.d	
*Cyperus articulatus*	Cyperaceae	Rhizomes	Methanol	NF54	4.8	n.d	Rukunga et al. (2008)
			Chloroform	NF54	2.1	n.d	
				ENT 30	3.3	n.d	
*Fagaropsis angolensis*	Rutaceae	Stem bark	Methanol	NF 54	4.68	brine shrimp nauplii 57.09 μg/mL	Kirira et al. (2006)
*Zanthoxylum usambarense*	Rutaceae	Stem bark	Methanol	NF 54	3.2	97.66 μg/mL	
*Suregada zanzibariensis*	Euphorbiaceae	Leaves	Methanol	D6	4.66	HELF cells >1000	Kigondu et al. (2009)
				W2	1.82		
*Schkuhria pinnata*	Asteraceae	Aerial	Pet ether	K1	2.46	>12.20 μg/mL	Obbo et al. (2019)
*V. lasiopus*	Asteraceae	Leaves	Chloroform	K39	1.2	n.d	Muregi et al. (2003)
			EtOAc	K39	1	n.d	
			Methanol	K39	3.2	n.d	
*Boscia salicifolia*	Rubiaceae	stem barks	water	D6	3.6	n.d	Muthaura et al. (2015a)
			methanol	D6	1.1	n.d	
		leaves	methanol	D6	4.4	n.d	
*Commiphora schimperi*	Burseraceae	stem barks	methanol	D6	3.9	n.d	
*Artemesia afra*	Asteraceae	leaves	water	W2	4.6	n.d	Muthaura et al. (2015b)
			Methanol	W2	3.9	n.d	
		stem barks	water	W2	4.1	n.d	
			Methanol	W2	1.2	n.d	
*Artemisia annua*	Asteraceae	Leaves	methanol	D6	4.7	n.d	
*Clerodendrum rotundifolium*	Verbenaceae	leaves	DCM	D6	3.9	n.d	
*Croton macrostachyus*	Euphorbiaceae	stem bark	methanol	D6	3.8	n.d	
*Cyperus articulatus*	Cyperaceae	tuber	methanol	D6	4.8	n.d	
*Fagaropsis angolensis*	Rutaceae	stem barks	methanol	D6	4.2	n.d	
*Hypoestes forskaolii*	Acanthaceae	root barks	methanol	D6	4.3	n.d	
*Maytenus heterophylla*	Celastraceae	Root barks	methanol	D6	1.8	n.d	
				W2	3.9	n.d	
*Maytenus obtusifolia*	Celastraceae	root bark	methanol	D6	<1.9	n.d	
*Parinari curatellifolia*	Chrysobalanaceae	root bark	methanol	W2	3.9	n.d	
*Rubia cordifolia*	Rubiaceae	whole plant	methanol	D6	<5	n.d	
				W2	<5	n.d	
*Stephania abbyssinica*	Menispermaceae	root barks	methanol	D6	4.7	n.d	
		leaves		D6	4.7	n.d	
*Turrea robusta*	Meliaceae	stem barks	methanol	D6	2.1	n.d	
*Warburgia ugandensis*	Canellaceae	root bark		W2	4.1	n.d	
*Zanthoxylum usambarense*	Rutaceae	root barks	methanol	D6	3.2	n.d	
*Schkuhria pinnata*	Compositae	Whole plant	methanol	D6	1.3	n.d	
*Clerodendrum eriophyllum*	Verbenaceae	Leaves	methanol	D6	1.8	n.d	
				W2	3.9	n.d	
*Fuerstia africana*	Lamiaceae	Whole plant	Methanol	D6	0.98	954.7 μg/mL	Muthaura et al. (2007b)
				W2	2.4		
*Schkuhria pinnata*	Asteraceae	whole plant	Methanol	D6	1.3	161.5 μg/mL	
*Boscia angustifolia*	Capparaceae	Leaves	water	D6	1.42	6720 μg/mL	
				W2	4.77		
*Boscia angustifolia*	Capparaceae	stem barks	water	D6	1.4	n.d	Muthaura et al. (2015a)
				W2	4.7	n.d	
*Ludwigia erecta*	Onagraceae	Leaves	Methanol	D6	4.1	VERO cells 544.3 μg/mL	Muthaura et al. (2007b)
			water	D6	0.93	3283.6 μg/mL	
				W2	1.61		
*Teclea nobilis*	Rutaceae	Stem barks	Methanol	D6	3.9	n.d	Muthaura et al. (2015b)
		root barks	Methanol	D6	4.5	n.d	
*Ludwigia erecta*	Onagraceae	whole plant	water	D6	0.9	n.d	
				W2	1.6	n.d	
			methanol	D6	4.1	n.d	
*Toddalia asiatica*	Rutaceae	Fruits	Ethyl acetate	W2	1.87	Vero 199 Cells >100 μg/mL	Orwa et al. (2013)
				D6	4.01	>100 μg/mL	
		Root bark	Methanol	W2	2.49	>100 μg/mL	
			Water	W2	2.43	>100 μg/mL	
				D6	1.98	>100 μg/mL	
		Leaves	Ethyl acetate	D6	2.72	>100 μg/mL	
*Fuerstia africana*	Lamiaceae	Whole plant	methanol	D6	0.9	n.d	Muthaura et al. (2015a)
				W2	2.4	n.d	
*Pentas lanceolata*	Rubiaceae	Aerial parts	Water	D6	3.744	≥100 μg/mL	Rotich et al. (2015)
*Fuerstia africana*	Lamiaceae	Aerial parts	Water	D6	1.84	≥100 μg/mL	
*Ximenia americana*	Olacaceae	Stem barks	Water	D6	2.108	≥100 μg/mL	
*Premna chrysoclada*	Verbenaceae	Stems	Methanol	D6	0.75	Vero E6 Cells >100/mL	Gathirwa et al. (2011)
*Flueggea virosa*	Euphorbiaceae	Leaves	methanol	D6	2.2	n.d	Muthaura et al. (2015a)
				W2	3.6	n.d	
*Turraea robusta*	Meliaceae	Root barks	Methanol	D6	2.09	24.38 μg/mL	Gathirwa et al. (2008)
*Turraea robusta*	Meliaceae	Root barks	Methanol	K1	3.5	n.d	Irungu et al. (2007)
				NF54	2.4		
*Turraea robusta*	Meliaceae	Stem barks	DCM: methanol	W2	2.87	VERO cells 21.9 μg/mL	Irungu et al. (2015)
				D6	2.3	4TI 5.3 μg/ml	
*Artemisia afra*	Asteraceae	Leaves	Methanol	W2	3.98	Vero cells 594.8 5 μg/mL	Gathirwa et al. (2007)
			Water	W2	4.65	2825.21 μg/mL	
*Boscia salicifolia*	Capparidaceae	Stem barks	Methanol	D6	1.04	304.92 μg/mL	
			Water	D6	3.65	1683.95 μg/mL	
*Catharanthus roseus*	Apocynaceae	Leaves	Methanol	D6	4.65	167.52 μg/mL	
			methanol	D6	4.6	n.d	Muthaura et al. (2015a)
*Clutia robusta*	Euphorbiaceae	leaves	methanol	D6	3.4	n.d	Muthaura et al. (2015a)
*Clutia robusta*	Euphorbiaceae	Leaves	Methanol	D6	3.41	460.29 μg/mL	Gathirwa et al. (2007)
*Rotheca myricoides*	Verbenaceae	root barks	methanol	D6	4.7	n.d	Muthaura et al. (2015a)
				W2	4.3	n.d	
*Acacia mellifera*	Leguminosae	Root barks	DCM	W2	4.2	n.d	Muthaura et al. (2015a)
		Leaves	Methanol	D6	3.9	n.d	
*Sericocomopsis hilde brandtii*	Amaranthacea	Aerial parts	Methanol	D6	3.15	≥100 μg/mL	Rotich et al. (2015)
				D6	4	≥100 μg/mL	
*Sericocomopsis hilde brandtii*	Amaranthacea	Root barks	Water	D6	2.12	≥100 μg/mL	Kigondu et al. (2011)
*Fuerstia africana*	Lamiaceae	Aerial parts	Pet ether	D6	1.56	n.d	
				W2	2.5		
		Roots	Pet ether	D6	4.6		
*Fuerstia africana*	Lamiaceae	Whole plant	methanol	D6	0.9	n.d	Muthaura et al. (2015a)
				W2	2.4	n.d	

DCM, dichloromethane; pet ether = Petroleum ether; K = *zanthoxylum chalybeum* collected from kilifi county kenya; T = *zanthoxylum chalybeum* collected from tharaka nithi county kenya; EtOAc, ethyl acetate; n. d = not done.

**TABLE 3:** Plant extracts with highest antimalarial activity (chemosuppression ≥50%).

Plant screened	Family	Part used	Solvent used	Parasite suppression (%) (dose)	LD_50_	References
*Premna chrysoclada*	Verbenaceae	Stems	Methanol	65.08 (250 mg/kg)	n.d	Gathirwa et al. (2011)
		Leaves	Methanol	65.08 (250 mg/kg)	n.d	
*Flueggea virosa*	Euphorbiaceae	Roots	Methanol	68.55 (250 mg/kg)	n.d	
*Azadirachta indica*	Meliaceae	Leaves	Methanol	89.16 (250 mg/kg)	n.d	
*Rhus natalensis*	Anacardiaceae	Leaves	Methanol	82.7 (250 mg/kg)	n.d	
*Grewia plagiophylla*	Tiliaceae	Leaves	Methanol	77.9 (250 mg/kg)	n.d	
*Hoslundia opposita*	Labietaceae	Roots	Methanol	79.67 (250 mg/kg)	n.d	
		Aerial parts	Methanol	55.05 (250 mg/kg)	n.d	
*Combretum padoides*	Combretaceae	Roots	Methanol	50.56 (250 mg/kg	n.d	
		Stem barks	Water	83.08 (250 mg/kg)	n.d	
			Methanol	91.37 (250 mg/kg)	n.d	
*Allophylus pervillei*	Sapindaceae	Stem barks	Methanol	62.1 (250 mg/kg)	n.d	
*Lannea schweinfurthii*	Anacardiaceae	stem barks	water	83.08 (100 mg/kg)	n.d	Gathirwa et al. (2008)
			methanol	91.37 (100 mg/kg)	n.d	
*Sclerocarya birrea*	Anacardiaceae	stem barks	water	66.51 (100 mg/kg)	n.d	
			methanol	63.49 (100 mg/kg)	n.d	
*Turraea robusta*	Meliaceae	Root barks	Water	63.8 (100 mg/kg)	n.d	
			Methanol	78.2 (100 mg/kg)	n.d	
*Artemisia afra*	Asteraceae	Leaves	Methanol	77.45 (100 mg/kg)	n.d	Gathirwa et al. (2007)
		Leaves	Water	70.25 (100 mg/kg)	n.d	
*Boscia salicifolia*	Capparidaceae	Stem barks	Methanol	86.5 (100 mg/kg)	n.d	
*Rhus natalensis*	Anacardiaceae	Stem barks	Methanol	56.24 (100 mg/kg)	n.d	
*Rhus natalensis*	Anacardiaceae	Stem barks	Water	83.15 (100 mg/kg)	n.d	
*Rotheca myricoides*	Verbenaceae	Leaves	Methanol	82.17 (800 mg/kg)	n.d	Jeruto et al. (2015)
*Rotheca myricoides*	Verbenaceae	Root barks	Methanol	61.18 (800 mg/kg)	n.d	
*Asparagus racemosus*	Asparagaceae	Leaves	Methanol	54.35 (800 mg/kg)	n.d	
*Pentas lanceolata*	Rubiaceae	Aerial parts	Methanol	64.9 (500 mg/kg)	>5000 mg/Kg	Rotich et al. (2015)
*Ximenia americana*	Olacaceae	Stem barks	Water	54.9 (500 mg/kg)	n.d	
			Methanol	50.8 (500 mg/kg)	n.d	
*Turraea mombassana*	Meliaceae	Leaves	Methanol	52.86 (800 mg/kg)	>5000 mg/kg	Nyangacha et al. (2012)
*Ludwigia erecta*	Onagraceae	Leaves	Methanol	65.28 (100 mg/kg)	>100 mg/kg	Muthaura et al. (2007b)
*Boscia angustifolia*	Capparaceae	Leaves	Methanol	60.12 (100 mg/kg)	>100 mg/kg	
*Pittosporum viridiflorum*	Pittosporaceae	Leaves	Methanol	54.77 (100 mg/kg)	>100 mg/kg	
			Water	89.76 (100 mg/kg)	1000 mg/kg	
*Clutia abyssinica*	Euphorbiaceae	Leaves	Water	71.69 (100 mg/kg)	>5000 mg/kg	
*Fuerstia africana*	Lamiaceae	Whole plant	Methanol	61.85 (100 mg/kg)	>100 mg/kg	
*Schkuhria pinnata*	Asteraceae	whole plant	Water	64.22 (100 mg/kg)	>5000 mg/kg	
*Clerodendrum eriophyllum*	Verbenaceae	Root bark	Methanol	90.13 (100 mg/kg)	>100 mg/kg	
			Water	61.54 (100 mg/kg)	>5000 mg/kg	
*Clausena anisata*	Rutaceae	Stem barks	Hexane	56.7 (500 mg/kg)	4166.7 mg/kg	Irungu et al. (2012)
			Chloroform	73.4 (500 mg/kg)	4166.7 mg/kg	

DCM, dichloromethane; pet ether *=* Petroleum ether; EtOAc, ethyl acetate; n. d = not done. Parasite strain for all *in vivo* studies: *Plasmodium berghei ANKA*

### 3.4 *In vitro* and *in vivo* activities of isolated compounds

Fifty five antimalarial/antiplasmodial active compounds isolated from eight plant species were reported. Of the 55 compounds, 7 (12.7%) and 48 (87.3%) were evaluated *in vivo* and *in vitro,* respectively. Twenty two of 55 (40%) compounds exhibited moderate activity while 16 (29%) were inactive. The most active compounds (IC_50_ values ≤10 μg/mL) were 17 (i.e., **5, 25, 26, 27, 28, 29, 31, 32, 34, 37, 40, 41, 42, 44, 46, 48**) (Table 4) with resinone (**39**) having the best activity (IC_50_ < 1 μg/mL).

**TABLE 4:** Compounds with highest antiplasmodial activity (IC_50_ ≤ 10 μg/mL).

Plant screened	Compound isolated	Class	Parasite strain	IC_50_	Cytotoxicity	References
*Turraea nilotica*	Azadironolide **(5)**	Terpenoid	D6	2.4 µM	4TI 14.7 μg/mL	Irungu et al. (2015)
			W2	1.1 µM	HEp2 8.5 μg/mL	
					Vero	
					27.6 μg/mL	
*Tephrosia elata*	Elatadihydrochalcone **(25)**	Flavonoids	D6	8.4 μg/mL		Muiva et al. (2009)
			W2	8.6 μg/mL		
			D6	2.8 μg/mL		
			W2	5.5 μg/mL		
	Acetoxyelatadihydrochalcone **(26)**		D6	9.6 μg/mL		
	Obovatin **(27)**		D6	4.9 μg/mL		
			W2	6.4 μg/mL		
	Obovatin methyl ether **(28)**		D6	3.8 μg/mL		
			W2	4.4 μg/mL		
	Deguelin **(29)**		D6	6.3 μg/mL		
			W2	8.9 μg/mL		
*Tephrosia subtriflora*	MS-II **(31)**	Flavanol	D6	4.6 µM	Vero >247.5 µM	Muiva-Mutisya et al. (2018)
			3D7	1.7 µM	HEp 2 > 247.5 µM	
			KSM	1.5 µM		
			F32-TEM	1.4 µM		
	Spinosaflavanone B **(32)**	Flavanone	D6	5.9 µM	n.d	
			3D7	5.5 µM	n.d	
			KSM	6.6 µM		
*Drypetes gerrardii*	Friedelin **(34)**	Terpenoids	K1	4.8 μg/mL	L6	Ng′ang′a et al. (2012)
					>90 μg/mL	
	5 β,24-cyclofriedelan-3-one **(37)**		K1	2.2 μg/mL	21.2 μg/mL	
	Resinone (**39)**		K1	0.09 μg/mL	84.8 μg/mL	
	β - Sitosterol glucopyranoside **(40)**		K1	5.4 μg/mL	14.3 μg/mL	
	Amentoflavone **(41)**		K1	2.6 μg/mL	0.34 μg/mL	
*Erythrina burtii*	Burttinol-A **(42)**	Isoflav-3-enes	D6	7.6 µM	n.d	Yenesew et al. (2012)
			W2	8.5 µM		
	Burttinol-C **(44)**		D6	9.3 µM		
			W2	9.1 µM		
	Burttinol-D **(46)**	2-Arylbenzofuran	D6	4.0 µM		
			W2	6.1 µM		
	Abyssinone V **(48)**	Flavanones	D6	5.7 µM		
			W2	6.6 µM		


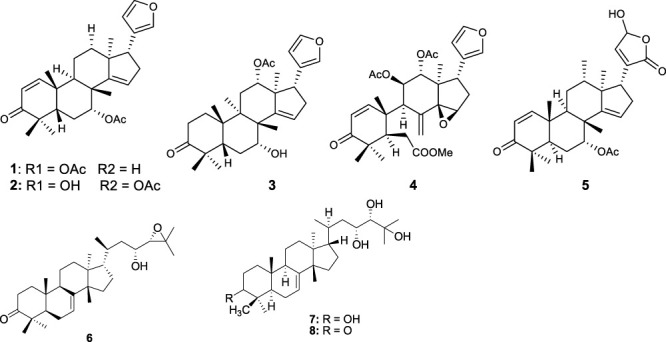



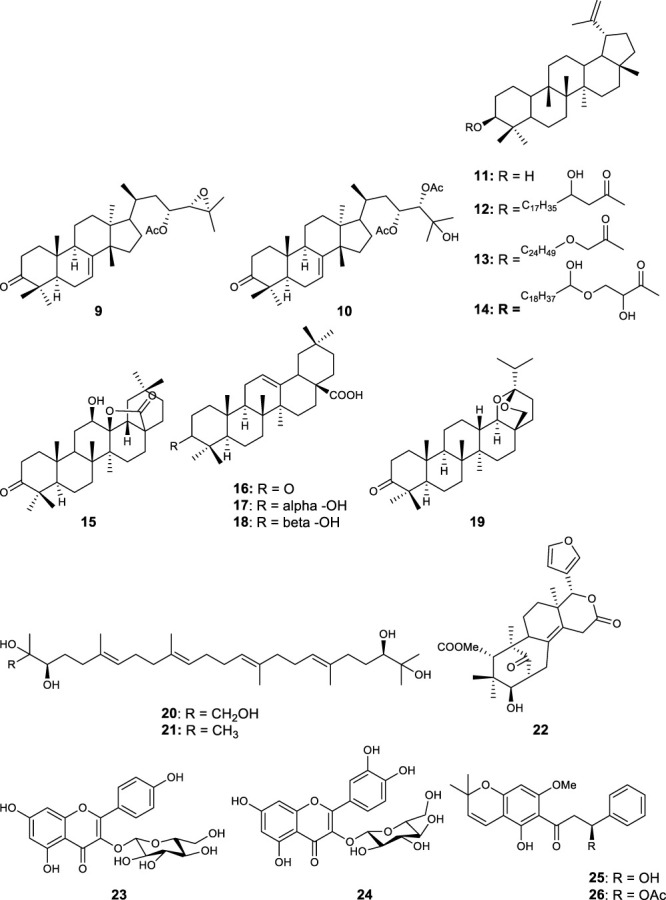



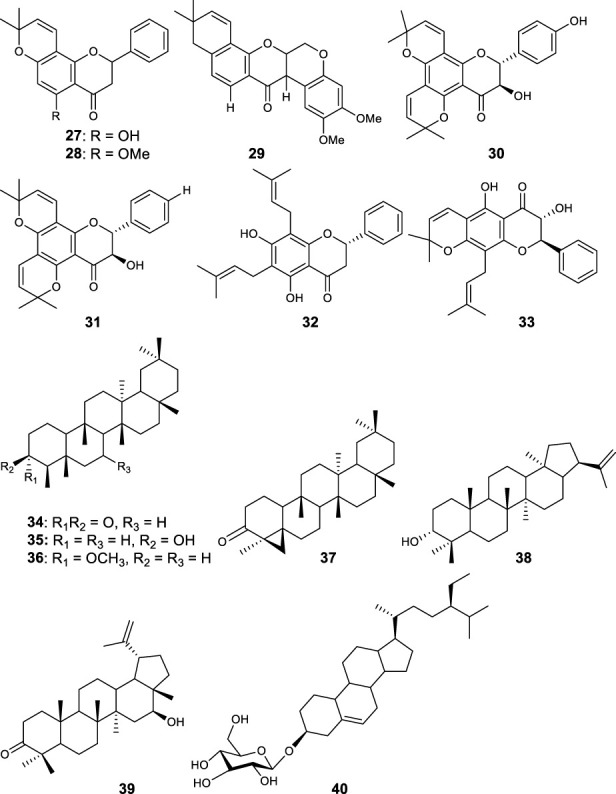



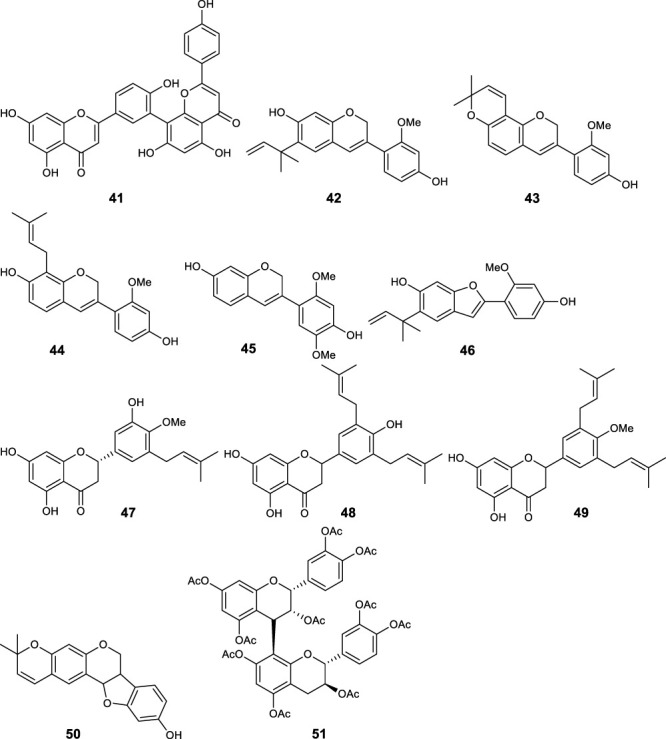



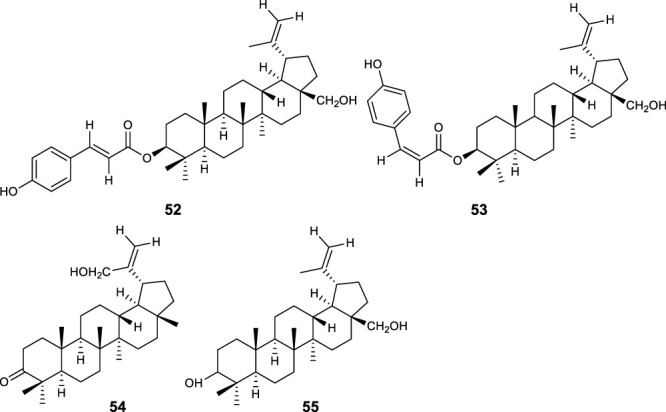


### 3.5 Cytotoxicity of plant extracts and compounds evaluated for antimalarial and antiplasmodial activity

In this review, a promising antimalarial extract was classified as lacking cytotoxicity to the mammalian cells by displaying an IC_50_ value greater than 90 μg/mL (Irungu et al., 2007). In general, there were 210 cytotoxicity tests from 40 plants. Out of the 40 plant species 14 (35%) had some degree of cytotoxicity across different studies. Plant families with the most cytotoxic (CC_50_ < 10 μg/mL) plant species were Meliaceae, Cucurbitaceae, Canellaceae, Asclepiadaceae, Asparagaceae and Lamiaceae. Fourteen (35%) of the plants tested were cytotoxic (CC_50_ < 10 μg/mL) and 8 (20%) demonstrated some toxicity levels (LD_50_ 100 mg/kg) in mice. The plants with good and moderate antiplasmodial activity demonstrated some degree of cytotoxicity of 10% and 7.5%, respectively. Organic extracts especially methanol, petroleum ether, dichloromethane: 1; 1 mixture of methanol and dichloromethane were reported to have the highest degree of cytotoxicity (CC_50_ < 10 μg/mL). The most cytotoxic was dichloromethane extract from *Warburgia ugandensis* Sprague with CC_50_ 0.34 μg/mL against L6, rat skeletal myoblast cells (Irungu et al., 2007). The most cytotoxic compounds were azadironolide (**5**) with CC_50_ of 8.5 μg/mL (HEp2 cells), oleanonic acid (**16**) with CC_50_ of 1.4 µM (HEp2 cells), 12α-acetoxy-7-deacetylazadirone (**2**) with CC_50_ of 4.3 µM (HEp2 cells), niloticin (**6**) with CC_50_ of 6.9 µM (HEp2 cells), hispidol B (**7**) with CC_50_ of 7.4 µM (HEp2 cells), amentoflavone (**41**) with CC_50_ of 0.34 μg/mL (L6 cells) and piscidinol A (**8**) with CC_50_ of 8.4 µM (HEp2 cells). The mentioned compounds had promising antiplasmodial activity, with azadironolide (**5**) being the most active. The most frequently used cells for cytotoxicity determination were Vero cells and HEp-2 cells.

## 4 Discussion

The global concern over the increasing resistance to primary antimalarial medications necessitates a boost in research efforts to discover and develop new drugs for malaria. The escalating resistance rates emphasize the urgency of accelerating the exploration and development of novel antimalarial drugs. It is evident from this review that a lot needs to be done towards the discovery of new antimalarial drugs. While numerous plant species have shown promising antiplasmodial effects, there has been limited evaluation of these plants in animal models, with only 9% (105/1170) *in vivo* studies and no clinical trial conducted. This highlights the importance of conducting comprehensive preclinical and clinical research. Pre-clinical and clinical research are a significant next step to determine the prospects of these promising medicinal plants (Al Rashid et al., 2020).

The majority of studies (91.6%) included in the analysis utilized crude plant extracts rather than pure compounds for their investigations. Such preference for crude extracts can be attributed to insufficient infrastructure required to process plant materials and extract pure compounds as well as an attempt to mimic the traditional preparation of plant remedies using alcoholic beverages. Preference for leaves, stem barks and root barks (Figure 2), can be attributed to their abundance and the local communities’ indigenous knowledge and skills on their uses (Umair et al., 2019). Additionally, the preference for harvesting these plant parts is influenced by their lower impact on the overall health and sustainability of medicinal plant populations (Araya et al., 2015).

In this review, the IC_50_ values below 10 μg/mL were regarded as the threshold for significant antimalarial activity. This cutoff is considered as the minimum requirement for preliminary positive result in screening of potential antimalarial plant extracts (Mohammed et al., 2014). A total of 151 plant species belonging to 48 families exhibited moderate to good antiplasmodial activity. Among the most extensively studied plant families were Asteraceae, Verbenaceae, Fabaceae, Euphorbiaceae, Rubiaceae, and Leguminosae while families with the highest number of active plants were Apocynaceae, Celestraceae, Euphorbiaceae and Rutaceae. These findings suggest that greater attention should be given to plants whose extracts were promising for the discovery of antimalarial drug leads. Regarding individual plant species, notable ones that have received significant research attention include *R. myricoides, A. indica, R*. *natalensis, T*. *robusta, X*. *americana, T*. *asiatica, M*. *undata, L. schweinfurthii, Z*. *chalybeum, H. abyssinica, F. africana*, *A*. *racemosus* and *T*. *robusta.* Their extracts have consistently demonstrated significant antiplasmodial activities in multiple studies (Gathirwa et al., 2007; 2008; 2011; Orwa et al., 2013; Muthaura et al., 2015a; Jeruto et al., 2015; Rotich et al., 2015). Therefore, further preclinical evaluation of these plant species is recommended. This review has identified most potent plant extracts with significant activity against *P*. *falciparum*, exhibiting an IC_50_ value of ≤1 μg/mL and/or a parasite suppression rate above 90%. The plant species whose extracts were classified as most potent include *Combretum padoides* Engl. and Diels, *L. schweinfurthii*, *Clerodendrum eriophyllum* (Hochst.) Vatke, *Holarrhena floribunda* (G. Don) T. Durand and Schinz, *M*. *undata*, *E*. *burtii*, *Vernonia lasiopus* O. Hoffm., *F*. *africana*, *Ludwigia erecta* (L.) H. Hara, *Boscia salicifolia* Oliv., and *Premna chrysoclada* (Bojer) Gürke. Furthermore, studies conducted by other researchers (Machumi, 2010; Machumi et al., 2010; Muganga et al., 2010; Zofou et al., 2013; Hoekou et al., 2017; Elhaj et al., 2021) have also reported good antiplasmodial activities of *C*. *eriophyllum*, *H*. *floribunda*, *V*. *lasiopus*, *F*. *africana*, and *L*. *erecta*.

In this review, we documented compounds that are reported to possess other pharmacological activities such as obovatin (**27**) which has shown great potential as an antibacterial agent (Akter et al., 2016). Other studies have demonstrated antiplasmodial and anticancer activities of compounds captured in this review, deguelin (**29**), (Varughese et al., 2019; Buyinza, 2020), friedelin (**34**) (Prabhu et al., 2011; Emsen et al., 2018; Joshi et al., 2022; Wuttikit and Thanakijcharoenpath, 2023), and epifriedelanol (**35**) (Kundu et al., 2000; Gashu, 2022; Wuttikit and Thanakijcharoenpath, 2023). The antiplasmodial activity of friedelin (**34**) was found to be lower in a study by Sadeghpour et al. (2006) compared to other research cited in the current review (Sadeghpour et al., 2006). In summary, these compounds have demonstrated promising antiplasmodial activity and are thus valuable candidates for further antimalarial drug development.

Our review has demonstrated that majority of investigated plants have promising antiplasmodial activity. However, when the same plants were tested in a mouse model, their activity against malaria parasites decreased in most cases, with many plants showing no activity at all. For instance, Rotich et al. (2015) and Gathirwa et al. (2011) reported that *Uvaria acuminate* Oliv. and *F*. *africana*, displayed good antiplasmodial activity (IC_50_ < 10 μg/mL) but were inactive *in vivo* (chemosupressiom at 27.0% and 27.9%, respectively). The observed variations could be explained by the fact that *in vitro* studies involved direct contact between the extracts and the parasite, while for *in vivo* studies activity of the extracts/compounds might have been altered by metabolism. Nevertheless, a few studies have shown that plant activity can actually increase from *in vitro* to *in vivo*. For example, Muthaura et al. (2007b) demonstrated that *Pittosporum viridiflorum* Sims exhibited moderate activity *in vitro* [IC_50_ 18.9 μg/mL and 17.7 μg/ml against D6 and W2 strains, respectively] but showed good activity *in vivo* with chemosuppression of 54.8% (Muthaura et al., 2007b). These findings suggest that plants could still possess significant antimalarial properties in animal models even if they do not show activity *in vitro*. Apparently, researchers proceed to *in vivo* studies only when they observe substantial antiplasmodial activity. This may explain the limited number of *in vivo* studies documented in this review. Despite the unsatisfactory outcomes observed *in vitro*, it still remains crucial to examine the antimalarial properties of plants through *in vivo* studies.

The present study identified significant inter study variations in the antiplasmodial activity of various plant species. Notably, considerable variation was observed for species such as *P*. *chrysoclada, F*. *virosa, Grewia plagiophylla* Burret*, T*. *robusta, R. myricoides, A*. *racemosus, Vangueria acutiloba* K. Schum.*, C. eriophyllum, H*. *abyssinica, V*. *lasiopus, W*. *ugandensis, Ajuga remota* Benth.*, Tabernaemontana pachysiphon* Stapf*, Uvaria lucida* Benth.*, Uvaria scheffleri* Engl. and Diels*, Vitex strickeri* Moldenke*, Warburgia stuhlmannii* Engl. and *Cyperus articulatus* L. (Muthaura et al., 2007a; Muthaura et al., 2015a; Muthaura et al., 2015b; Irungu et al., 2007; Gathirwa et al., 2008; Gathirwa et al., 2011; Rukunga et al., 2008; Jeruto et al., 2015). Several factors may account for these differences, including variations in the extraction solvent used, which affects the yield and composition of extracted metabolites. Dichloromethane, for instance, primarily extracts apolar metabolites, while methanol extracts a range of polar to moderately apolar metabolites and water extracts polar metabolites. The choice of plant parts used in the studies also contributed to the observed variations, as certain parts may contain higher concentrations of specific active metabolites. Additionally, differences in extraction yields can arise due to the varying accumulation of active metabolites in different plant parts. Also, the location, environmental factors and season (dry and rainy seasons) have significant effect on the accumulation of various phytochemicals present in medicinal plants. During the dry season, there is a decrease in water and nutrient supply to plants. Nutritional stress can result in the accumulation of osmo-protectants to stabilize proteins structure and maintain membrane integrity and scavenge reactive oxygen species (ROS), with biomass and secondary metabolites production (Niinemets, 2016). Phenolic compounds including coumarins, flavonoids, cinnamic acids and lignans, as well as plant hormones such as auxins, salicylic acid, cytokinin, ethylene, gibberellic acid and jasmonic acid are involved in modulation of developmental processes in plants and determine plant responses to environmental stresses (Fang et al., 2011; Fayez and Bazaid, 2014; Giménez et al., 2014). On the other hand, plants growing in lower temperatures develop significant adjustments in several physiological and biochemical processes that enable them to survive under low temperature stress, and this causes inhibition in the synthesis and storage of secondary metabolites (Verma and Shukla, 2015).

Another factor that may contribute to the observed inter study variation is the strain of *Plasmodium* used in the experiments. Studies employing chloroquine-sensitive strains of the parasite, such as *P. falciparum* 3D7, D6, and NF54, tend to report higher antiplasmodial activity compared to studies utilizing chloroquine-resistant strains like W2, K39, ENT30, or K1. This variation in strain susceptibility to the tested extract/compound can influence the reported outcomes and contribute to the differences observed across studies. It is worth noting that the variation in the antiplasmodial activity of *Turraea nilotica* Hochst. ex Benth (Irungu et al., 2015). observed with pure compounds highlights an important issue. Even extracts that initially show low potency and might be disregarded during the initial screening process for further development may still contain active components with therapeutic potential, as mentioned by Kuria et al. (2001). In the given example, the preliminary analysis of the crude extract demonstrates an IC_50_ value of 59 μg/mL for the D6 strain and 47.4 μg/mL for the W2 strain, as indicated in Supplementary Table S1. However, within the same extract, there is a highly activeepimeric mixture, azadironolide (**51**) that exhibited an IC_50_ value of less than 5 μg/mL.

Data collated in this review showed 14 out of 40 (35%) plant species, exhibited high level of cytotoxicity (CC_50_ < 10 μg/mL). The plant families Meliaceae, Cucurbitaceae, Asclepiadaceae, Asparagaceae, Canellaceae and Lamiaceae were found to have the highest number of cytotoxic plant species. The most cytotoxic plants identified were *W*. *ugandensis, X*. *americana* and *Khaya anthotheca* (Welw.). Interestingly, *W*. *ugandensis* and *X*. *americana* have shown promising antiplasmodial/antimalarial activity in certain studies (Irungu et al., 2007; Muthaura et al., 2015b). This suggests that the observed strong antiplasmodial effects could probably be as a result of cytotoxicity rather than direct activity against the parasites themselves (Irungu et al., 2007). Other plants with significant cytotoxicity but also exhibiting moderate to good antiplasmodial/antimalarial properties include *Vernonia amygdalina* Delile, *Baccharoides adoensis* (Sch.Bip. ex A. Rich.) Hochr., *Schkuhria pinnata* (Lam.) Kuntze, *Momordica foetida* Schumach. and Thonn., *Entada abyssinica* Steud. ex A. Rich., *Entandrophragma utile* (Dawe and Sprague) Sprague (Obbo et al., 2019), *C*. *eriophyllum* (Irungu et al., 2007), *Ekebergia capensis* Sparrm (Irungu et al., 2014), *T*. *robusta* (Irungu et al., 2015) and *F*. *africana* (Rotich et al., 2015). The toxicity levels of most plant extracts in animal models were found to be minimal, even at dosages above 1000 mg/kg body weight. Aqueous extracts showed no adverse effects even at a dosage of 5000 mg/kg body weight. It is important to note that toxicity/cytotoxicity levels varied considerably, even within the same plant species. This variation could be attributed not only to the extraction solvent but also to differences in study design (*in vivo* or *in vitro*) and the specific plant parts tested.

## 5 Conclusion

This review has collated valuable foundational data that researchers in the field can utilize for the exploration and development of new antimalarial drug leads. Among the plant species studied, *F*. *africana* and *L*. *erecta* were found to have the highest activity, with IC_50_ values below 1 μg/mL against *P*. *falciparum* (D6), a chloroquine-sensitive strain. These plants also demonstrated significant parasite suppression at an oral dose of 100 mg/kg, with 61.9% and 65.3% for *F*. *africana* and *L*. *erecta*, respectively. Their LD_50_ values were above 3000 mg/kg, indicating low toxicity. Additionally, resinone (**39**) a compound isolated from the *Drypetes gerrardii* (Baill.) Hutch showed good activity against *P. falciparum* K1 multidrug-resistant strain, with an IC_50_ below 1 μg/mL. However, no information was provided regarding *in vivo* testing or toxicity assessments of this compound. While the *in vitro* results demonstrated promising activities of some plant extracts and their compounds, there has been limited evaluation of active plants extracts *in vivo*, and no clinical trials have been conducted yet. To address the research gap, preclinical studies should progress beyond *in vitro* and *in vivo* screening for antimalarial properties to include comprehensive studies on efficacy, safety and quality of promising extracts in animal models. Additionally, future studies geared towards product development should factor in intellectual property rights through local bodies such as Kenya Industrial Property Institute to address barriers that may arise and hinder development of lead compounds/phytomedicines from medicinal plants. Furthermore, the study revealed significant variations in the antiplasmodial activities of the plants across different studies. Notably, only a small number of plants had their active compounds identified. Furthermore, it is worth emphasizing the significance of assessing ethnomedical preparation procedures and establishing a correlation with laboratory extraction methods. This correlation is essential as it justifies the process of plant selection and, in turn, contributes to the validation of ethnomedicine. Hence, there is still need for further and extensive research with the aid of a stable strategy in the exploration and advancement of novel antimalarial compounds to tackle the escalating resistance observed in current primary antimalarial drugs across the globe.

## Abbreviations

KEMRI, Kenya Medical Research Institute; CTMDR, Center for Traditional Medicine and Drug Research; PRISMA, Preferred Reporting Items for Systematic Reviews and Meta-Analysis; WHO, World Health Organization; DNMP, Division of National Malaria Program; ICF, International Classification of Functioning disability, and health; IPTp, Intermittent Preventive Treatment during pregnancy; ACT, Artemisinin Combination Therapy; DCM, Dichloromethane; Pet ether, Petroleum ether; EtOAc, ethyl acetate.
