# Supplementary material for: Potential of medicinal plants as antimalarial agents: a review of work done at Kenya Medical Research Institute
Source: Front Pharmacol. 2023 Oct 20;14:1268924. doi: 10.3389/fphar.2023.1268924 (PMC10623325; doi:10.3389/fphar.2023.1268924)
Supplement: Supplementary file 1 [file DataSheet1.ZIP › Table 1.DOCX]

**Table S1 *In vitro* antiplasmodial activity of medicinal plants screened at KEMRI-CTMDR**

| **Plant screened** | **Plant Family** | **part used** | **Solvent used** | **Parasite strain** | **IC_50_ ug/ml** | **Activity** | **Cytotoxicity/LD _50_** | **Reference** |
| --- | --- | --- | --- | --- | --- | --- | --- | --- |
| *Aganthesanthemum bojeri* | Rubiaceae | Whole plant | Methanol | D6 | 55.3 | Inactive |  | (Gathirwa et al., 2011) |
|  |  |  |  | W2 | 55.97 | Inactive |  |  |
| *Abrus precatorius* | Leguminosae/Fabaceae | Whole plant | Methanol | D6 | 85.59 | Inactive |  |  |
|  |  |  |  | W2 | 100 | Inactive |  |  |
| *Turraea nilotica* | Meliaceae | Stem barks | DCM : Methanol | D6 | 7.3 | Good | Vero 17.7 µg/ml | (Irungu et al., 2015) |
|  |  | Root barks | DCM : Methanol | D6 | 9.5 | Good | Vero 13.7 µg/ml |  |
|  |  |  |  | W2 | 7.9 | Good | 4TI 18.6 µg/ml |  |
|  |  | Leaves | DCM : Methanol | D6 | 59 | Inactive | Vero 21.5 µg/ml |  |
|  |  |  |  | W2 | 47.4 | Inactive | 4TI 39.1 µg/ml |  |
| *Holarrhena floribunda* | Apocynaceae | Stem bark | Aqueous extract | W2 | 1.02 | Good | n.d | (Fotie et al., 2006) |
|  |  |  |  | D6 | 5.91 | Good | n.d |  |
|  |  |  | Ethanoic extract | W2 | 4.33 | Good | n.d |  |
|  |  |  |  | D6 | 5.22 | Good | n.d |  |
|  |  |  | Chloroform | D6 | 2.29 | Good | n.d |  |
|  |  |  |  | W2 | 18.53 | Moderate | n.d |  |
| *Ekebergia capensis* | Meliaceae | Roots | DCM : Methanol | D6 | 18.2 | Moderate |  | (Irungu et al., 2014) |
|  |  |  |  | W2 | 34 | Moderate | Vero 2.8 µg/ml |  |
|  |  |  |  |  |  |  | 4TI 9.3 µg/ml |  |
|  |  |  |  |  |  |  | HEp2 61 µg/ml |  |
|  |  | Leaves | DCM : Methanol | D6 | 44.9 | Moderate | MDA-MB-231 nd |  |
|  |  |  |  | W2 | 45.3 | Moderate | Vero 97.8 µg/ml |  |
|  |  |  |  |  |  |  | 4TI 82.1 µg/ml |  |
| *Caesalpinia volkensii* | Leguminosae | Leaves | Water | K1 | 68.7 | Inactive |  | (Irungu et al., 2007) |
|  |  |  |  | NF54 | 100 | Inactive | L 6 cells |  |
|  |  |  | Methanol | K1 | 51.4 | Inactive |  |  |
|  |  |  |  | NF54 | 65.1 | Inactive |  |  |
|  |  |  | DCM | K1 | 25.6 | Inactive |  |  |
|  |  |  |  | NF54 | 11.9 | Inactive | 82.4 µg/ml |  |
| *Centella asiatica* | Umbelliferae | Whole plant | Methanol | K1 | 19.9 | Moderate |  |  |
|  |  |  |  | NF54 | 26 | Moderate |  |  |
|  |  |  | DCM | K1 | 15.4 | Moderate |  |  |
|  |  |  |  | NF54 | 14.9 | Moderate |  |  |
| *Clerodendrum eriophyllum* | Verbenaceae | Root barks | Water | K1 | 64 | Inactive |  |  |
|  |  |  |  | NF54 | 94.3 | Inactive | 82.6 µg/ml |  |
|  |  |  | Methanol | K1 | 48.2 | Moderate |  |  |
|  |  |  |  | NF54 | 51.5 | Inactive |  |  |
|  |  |  | DCM | K1 | 15.8 | Moderate |  |  |
|  |  |  |  | NF54 | 10.9 | Moderate | 7.9 µg/ml |  |
| *Gompocarpus semilunatus* | Asclepiadaceae | Whole plant | Methanol | K1 | 34.4 | Moderate | >90 µg/ml |  |
|  |  |  |  | NF54 | 46.1 | Moderate |  |  |
| *Gymnema sylvestre* | Asclepiadaceae | Climbers | Methanol | W2 | 69.3 | Inactive | n.d | (Muthaura et al., 2015a) |
|  |  | Whole plant | Water | K1 | 100 | Inactive | n.d |  |
|  |  |  |  | NF54 | 100 | Inactive | n.d |  |
|  |  |  | Methanol | K1 | 58.4 | Inactive | n.d |  |
|  |  |  |  | NF54 | 40.8 | Moderate | n.d |  |
| *Harrisonia abyssinica* | Simaroubaceae | Stem barks | Water | K1 | 91.1 | Inactive | n.d |  |
|  |  |  |  | NF54 | 100 | Inactive | n.d |  |
|  |  |  | Methanol | K1 | 52.3 | Inactive | n.d |  |
|  |  |  |  | NF54 | 55.4 | Inactive | n.d |  |
|  |  |  | DCM | K1 | 4.4 | Good | n.d |  |
|  |  |  |  | NF54 | 5.6 | Good | n.d |  |
| *Pentas bussei* | Rubiaceae | Whole plant | Water | K1 | 100 | Inactive | n.d |  |
|  |  |  |  | NF54 | 100 | Inactive | n.d |  |
|  |  |  | Methanol | K1 | 35.2 | Moderate | n.d |  |
|  |  |  |  | NF54 | 40.5 | Moderate | n.d |  |
| *Strychnos heningsii* | Leguminosae | Twigs | Water | K1 | 29.6 | Moderate | n.d |  |
|  |  |  |  | NF54 | 33.7 | Moderate | n.d |  |
|  |  |  | Methanol | K1 | 14.6 | Moderate | n.d |  |
|  |  |  |  | NF54 | 17.9 | Moderate | n.d |  |
|  |  |  | DCM | K1 | 35.2 | Moderate | n.d |  |
|  |  |  |  | NF54 | 33.3 | Moderate | n.d |  |
| *Tarena greveolens* | Rubiaceae | Stem barks | Water | K1 | 100 | Inactive | n.d |  |
|  |  |  |  | NF54 | 100 | Inactive | n.d |  |
|  |  |  | Methanol | K1 | 40.7 | Moderate | n.d |  |
|  |  |  |  | NF54 | 52.6 | Inactive | n.d |  |
| *Vernonia auriculifera* | Asteraceae | Leaves | Water | K1 | 84.5 | Inactive | >90 µg/ml | (Irungu et al., 2007) |
|  |  |  |  | NF54 | 100 | Inactive |  |  |
|  |  |  | Methanol | K1 | 53.8 | Inactive |  |  |
|  |  |  |  | NF54 | 60.8 | Inactive |  |  |
|  |  |  | DCM | K1 | 32.7 | Moderate |  |  |
|  |  |  |  | NF54 | 27.3 | Moderate |  |  |
| *Vernonia lasiopus* | Asteraceae | Root barks | Water | K1 | 52.2 | Inactive |  |  |
|  |  |  |  | NF54 | 100 | Inactive |  |  |
|  |  |  | Methanol | K1 | 31.2 | Moderate |  |  |
|  |  |  |  | NF54 | 50.5 | Inactive |  |  |
|  |  |  | DCM | K1 | 4.7 | Good | > 90 |  |
|  |  |  |  | NF54 | 4.9 | Good |  |  |
| *Warbugia ugandensis* | Canellaceae | Stem barks | Water | K1 | 31.8 | Moderate | 14.3 µg/ml |  |
|  |  |  | Methanol | NF54 | 64 | Inactive |  |  |
|  |  |  |  | K1 | 17.8 | Moderate |  |  |
|  |  |  | DCM | NF54 | 24.3 | Moderate |  |  |
|  |  |  |  | K1 | 1.4 | Good | 0.34 µg/ml |  |
|  |  |  |  | NF54 | 2.2 | Good |  |  |
| *Leucas. calostachys* | Lamiaceae | Leaves | Chloroform | D6 | 40.2 | Moderate | n.d | (Jeruto et al., 2015) |
|  |  |  | Methanol | D6 | 88.4 | Inactive | n.d |  |
|  |  |  | Water | D6 | 100 | Inactive | n.d |  |
| *Ajuga remota* | Lamiaceae | Leaves | Methanol | D6 | 22.1 | Moderate | n.d |  |
|  |  |  | Chloroform | D6 | 100 | Inactive | n.d |  |
|  |  |  | Water | D6 | 100 | Inactive | n.d |  |
| *Carpobrotus edulis* | Aizoaceae | Roots | Methanol | D6 | 100 | Inactive | n.d |  |
|  |  |  | Chloroform | D6 | 100 | Inactive | n.d |  |
|  |  |  | Water | D6 | 100 | Inactive | n.d |  |
| *Spermacoce princeae* | Rubiaceae | Roots | Methanol | D6 | 100 | Inactive | n.d |  |
|  |  |  | Chloroform | D6 | 100 | Inactive | n.d |  |
|  |  |  | Water | D6 | 100 | Inactive | n.d |  |
| *Acacia tortilis* | Fabaceae | Seeds | Pet ether | D6 | 100 | Inactive |  | (E. V. M. Kigondu et al., 2011) |
|  |  |  | EtOAc | W2 | 100 | Inactive |  |  |
| *Manilkara discolor* | Sapotaceae | Leaves | Methanol | D6 | 11.52 | Moderate |  |  |
|  |  |  |  | W2 | 69.2 | Moderate |  |  |
|  |  |  | Pet ether | D6 | 30.4 | Moderate |  |  |
|  |  |  |  | W2 | 90.6 | Inactive | Vero cells >100 µg/ml |  |
|  |  |  | EtOAc | D6 | 42.6 | Moderate |  |  |
|  |  |  |  | W2 | 49.9 | Moderate |  |  |
|  |  | Stem barks | Methanol | D6 | 100 | Inactive | >500 µg/ml |  |
|  |  |  |  | W2 | 100 | Inactive | 68.37 µg/ml |  |
|  |  |  | Pet ether | D6 | 80.6 | Inactive |  |  |
|  |  |  |  | W2 | 69.4 | Inactive | 64.72 µg/ml |  |
|  |  |  | EtOAc | D6 | 23.6 | Moderate |  |  |
|  |  |  |  | W2 | 33.4 | Moderate |  |  |
| *Sericocomopsis Hildebrandhi* | Amaranthaceae | Roots | Methanol | W2 | ˃50 | Inactive | >500 µg/ml |  |
|  |  |  | Pet ether | W2 | 38.9 | Moderate | 367.1µg/ml |  |
|  |  |  | EtOAc |  |  |  | 375.3µg/ml |  |
|  |  | Aerial parts | Methanol | W2 | ˃50 | Inactive | 93.97µg/ml |  |
|  |  |  | Pet ether | W2 | 31.55 | Moderate | >500 µg/ml |  |
|  |  |  | EtOAc | W2 | 16.51 | Moderate | >500 µg/ml |  |
| *Tephrosia elata* | Leguminosae | Stem barks | Water | D6 | 100 | Inactive |  | (Rotich et al., 2015) |
|  |  |  |  | W2 | 100 | Inactive |  |  |
|  |  |  | Methanol | D6 | 70.33 | Inactive |  |  |
|  |  |  |  | W2 | 73.6 | Inactive | ND |  |
|  |  |  | Water | D6 | 100 | Inactive | ND |  |
|  |  |  |  | W2 | 100 | Inactive |  |  |
|  |  |  |  | D6 | 100 | Inactive |  |  |
|  |  |  |  | W2 | 89.2 | Inactive |  |  |
|  |  | Leaves | Methanol | D6 | 100 | Inactive | >248.7 µM |  |
|  |  |  |  | W2 | 13.8 | Moderate | >248.7 µM |  |
|  |  |  | Water | D6 | 49.5 | Moderate |  |  |
|  |  |  |  | W2 | 100 | Moderate |  |  |
| *Sericocomopsis hildebrandhi* | Amaranthacea | Aerial parts | Water | D6 | 18.004 | Moderate | ≥100 | (Rotich et al., 2015) |
|  |  |  |  | W2 | 78.695 | Inactive |  |  |
|  |  |  | Methanol | D6 | 3.153 | good | ≥100 |  |
|  |  |  |  | W2 | 12.688 | Moderate |  |  |
|  |  | roots | Water | D6 | 4.096 | good |  |  |
|  |  |  |  | W2 | 54.166 | Inactive |  |  |
|  |  |  | Methanol | D6 | 2.12 | good |  |  |
|  |  |  |  | W2 | 14.851 | Moderate |  |  |
| *Gymnema sylvest* | Asclepiadaceae | Climbers | Methanol | W2 | 69.3 | Inactive | n.d | (Muthaura et al., 2015a) |
| *Grewia plagiophylla* | Tiliaceae | Leaves | Methanol | D6 | 19.2 | Moderate | n.d | (Muthaura et al., 2015b) |
|  |  |  |  | W2 | 34.2 | Moderate | n.d |  |
|  |  | Leaves | Methanol | D6 | 13.2 | Moderate | n.d | (Muthaura et al., 2015a) |
|  |  |  |  | W2 | 34.2 | Moderate | n.d |  |
| *Harrisonia abyssinica* | Simaroubaceae | root barks | Methanol | D6 | 7.8 | good | n.d | (Muthaura et al., 2015a) |
|  |  |  |  | W2 | 11.4 | Moderate | n.d |  |
|  |  | Leaves | Methanol | D6 | 7.8 | Good |  | (Muthaura et al., 2007) |
|  |  |  |  | W2 | 11.4 | Moderate |  |  |
| *Harungana madagascariensis* | Guttiferae | Leaves | Water | D6 | ˃100 | Moderate | n.d | (Muthaura et al., 2015a) |
|  |  |  |  | W2 | ˃100 | Moderate | n.d |  |
|  |  |  | Methanol | D6 | 39.1 | Moderate | n.d |  |
|  |  |  |  | W2 | 43.7 | Moderate | n.d |  |
|  |  | Leaves | Water | D6 | 100 | Inactive |  | (Muthaura et al., 2007) |
|  |  |  |  | W2 | 100 | Inactive |  |  |
|  |  |  | Methanol | D6 | 39.1 | Moderate |  |  |
|  |  |  |  | W2 | 43.7 | Moderate |  |  |
| *Hoslundia opposita* | Labiatae | Leaves | Methanol | D6 | 15.2 | Moderate | n.d | (Muthaura et al., 2015a) |
|  |  |  |  | W2 | 25.6 | Moderate | n.d |  |
| *Hugonia castaneifolia* | Linaceae | Twigs | Water | W2 | 152.1 | Inactive | n.d |  |
|  |  |  | Methanol | D6 | 23.4 | Moderate | n.d |  |
| *Maytenus undata* | Celastraceae | Leaves | Water | D6 | 0.95 | Good | n.d |  |
|  |  |  |  | W2 | 12 | Moderate | n.d |  |
|  |  |  | Methanol | D6 | 7.4 | Good | n.d |  |
|  |  |  |  | W2 | 9.8 | Good | n.d |  |
|  |  | Root barks | Water | D6 | 5.5 | Good | n.d |  |
|  |  |  |  | W2 | 7.9 | Good | n.d |  |
|  |  |  | Methanol | D6 | 5.1 | Good | n.d |  |
|  |  |  |  | W2 | 4.9 | Good | n.d |  |
|  |  |  | Water | D6 | 100 | Moderate | n.d |  |
|  |  |  |  | W2 | 100 | Moderate | n.d |  |
|  |  |  | Methanol | D6 | 4.4 | Good | n.d |  |
|  |  |  |  | W2 | 10.2 | Moderate | n.d |  |
|  |  |  | Water | D6 | 5.6 | Good | n.d |  |
|  |  |  |  | W2 | 8.2 | Good | n.d |  |
| *Maytenus senegalensis* | Celastraceae | Root barks | Water | D6 | 100 | Inactive | n.d |  |
|  |  |  |  | W2 | 100 | Inactive | n.d |  |
|  |  |  | Methanol | D6 | 4.7 | Good | n.d |  |
|  |  |  |  | W2 | 9.8 | Good | n.d |  |
| *Mangifera indica* | Anacardiaceae | Stem barks | Methanol | D6 | 25 | Moderate | n.d |  |
| *Moringa oleifera* | Moringaceae | Leaves | Methanol | D6 | 9.8 | Good | n.d |  |
| *Ocimum basilcum* | Lamiaceae | Leaves | Methanol | D6 | 16.4 | Moderate | n.d |  |
| *Ocimum gratissimum* | Lamiaceae | Leaves | Methanol | D6 | 5.9 | Good | n.d |  |
|  |  | Root barks |  | D6 | 23.3 | Moderate | n.d |  |
|  |  |  |  | W2 | 27.9 | Moderate | n.d |  |
| *Pentas longiflora* | Rubiaceae) | root barks | Methanol | D6 | 13.3 | Moderate | n.d |  |
| *Premna chrysoclada* | Verbenaceae) | Leaves | Methanol | D6 | 11.1 | Moderate | n.d |  |
| *Pentas bussei* | Rubiaceae | Leaves | Methanol | D6 | 13.3 | Moderate | n.d |  |
| *Ricinus communis* | Euphorbiaceae | Leaves | Methanol | D6 | <25 | Moderate | n.d |  |
|  |  | Fruits |  | D6 | ˃25 | Moderate | n.d |  |
| *Rauvolfia mombasiana* | Apocynaceae | Root barks | Water | W2 | 100 | Inactive | n.d |  |
|  |  |  | Methanol | W2 | 9.1 | Good | n.d |  |
| *Schizozygia coffaeoides* | Apocynaceae | Leaves | Methanol | W2 | 10.5 | Moderate | n.d |  |
| *Scolopia zeyheri* | Flacourtiaceae | Leaves | Methanol | W2 | 24.9 | Moderate | n.d |  |
|  |  | Root barks | Water | D6 | 100 | Inactive | n.d |  |
|  |  |  |  | W2 | 100 | Inactive | n.d |  |
|  |  |  | Methanol | D6 | 100 | Inactive | n.d |  |
|  |  |  |  | W2 | 100 | Inactive | n.d |  |
| *Suregada zanzibarensis* | Euphorbiaceae | Leaves | Methanol | D6 | 6.7 | Good | n.d |  |
|  |  |  |  | W2 | 5.8 | Good | n.d |  |
| *Tabernaemontana pachysiphon* | Apocynaceae | Fruits | Water | D6 | 4.8 | Good | n.d |  |
|  |  |  |  | W2 | 3.4 | Good | n.d |  |
|  |  |  | Methanol | D6 | 3.9 | Good | n.d |  |
|  |  |  |  | W2 | 53.7 | Inactive | n.d |  |
|  |  | Leaves | Water | D6 | 25.3 | Moderate | n.d |  |
|  |  |  |  | W2 | 70.8 | Inactive | n.d |  |
|  |  |  | Methanol | D6 | 14.7 | Moderate | n.d |  |
|  |  |  |  | W2 | 25.4 | Moderate | n.d |  |
| *Terminalia spinose* | Combretaceae | Root barks | Water | D6 | 62.9 | Inactive | n.d |  |
|  |  |  | Methanol | D6 | 7.9 | Good | n.d |  |
| *Turraea floribunda* | Meliaceae | Stem barks | Methanol | W2 | 5.5 | Good | n.d |  |
| *Tridax procumbens* | Asteraceae | Whole plant | Water | W2 | 100 | Inactive | n.d |  |
|  |  |  | Methanol | W2 | 15.4 | Inactive | n.d |  |
|  |  |  | Water | W2 | 13.9 | Moderate | n.d |  |
| *Tamarindus indica* | Fabaceae | Root barks | Methanol | W2 | 35.2 | Moderate | n.d |  |
| *Uvaria lucida* | Annonaceae | Leaves | Water | D6 | 100 | Inactive | n.d |  |
|  |  |  |  | W2 | 100 | Inactive | n.d |  |
|  |  |  | Methanol | D6 | 5.9 | Good | n.d |  |
|  |  |  |  | W2 | 10.3 | Moderate | n.d |  |
| *Uvaria scheffleri* | Annonaceae | Leaves | Water | D6 | 97.2 | Inactive | n.d |  |
|  |  |  | Methanol | W2 | 6.8 | Good | n.d |  |
|  |  | Root barks | Water | D6 | 58.9 | Inactive | n.d |  |
|  |  |  | Methanol | W2 | 8.9 | Good | n.d |  |
| *Vernonia amygdalina* | Asteraceae | Leaves | Water | W2 | 3.8 | Good | n.d |  |
|  |  |  | Methanol | D6 | 4.9 | Good | n.d |  |
|  |  |  |  | W2 | 7.2 | Good | n.d |  |
| *Vitex strickeri* | Verbenaceae | Leaves | Water | D6 | 100 | Inactive | n.d |  |
|  |  |  |  | W2 | 100 | Inactive | n.d |  |
|  |  |  | Methanol | D6 | 26.7 | Moderate | n.d |  |
|  |  |  |  | W2 | 24.8 | Moderate | n.d |  |
| *Warburgia stuhlmannii* | Canellaceae | Stem barks | Water | D6 | 100 | Inactive | n.d |  |
|  |  |  |  | W2 | 100 | Inactive | n.d |  |
|  |  |  | Methanol | D6 | 1.8 | Good | n.d |  |
|  |  |  |  | W2 | 2.3 | Good | n.d |  |
| *Zehneria scabra* | Cucurbitaceae | Whole plant | Methanol | W2 | 1.8 | Good | n.d |  |
| *Ziziphus mucronata* | Rhamnaceae | Leaves | Water | D6 | 100 | Inactive | n.d |  |
|  |  |  | Methanol | W2 | 21.9 | Moderate | n.d |  |
| *Maytenus putterlickioides* | (Celastraceae) | Root barks | Water | D6 | ˃100 | Inactive | n.d |  |
|  |  |  |  | W2 | ˃100 | Inactive | n.d |  |
|  |  |  | Methanol | D6 | 4.4 | Good | n.d |  |
|  |  |  |  | W2 | 10.2 | Moderate | n.d |  |
| *Zanthoxylum chalybeum* | Rutaceae | Root barks | Water | D6 | 5.3 | Good | n.d |  |
|  |  |  |  | W2 | 3.1 | Good | n.d |  |
|  |  |  | Methanol | D6 | 3.7 | Good | n.d |  |
|  |  |  |  | W2 | 1.1 | Good | n.d |  |
| *Zanthoxylum chalybeum* | Rutaceae | Stem barks | Water | NF54 | 5.52 | Good | n.d | (Rukunga et al., 2009) |
|  |  |  |  | ENT30 | 2.32 | Good | n.d |  |
|  |  |  | Methanol | NF54 | 5.3 | Good | n.d |  |
|  |  |  |  | ENT30 | 3.14 | Good | n.d |  |
|  |  |  | Water | NF54 | 3.65 | Good | n.d |  |
|  |  |  |  | ENT30 | 2.88 | Good | n.d |  |
| *Teclea simplicifolia* | Rutaceae | Stem barks | Water | NF54 | 80.43 | Inactive | n.d |  |
|  |  |  |  | ENT30 | 96.56 | Inactive | n.d |  |
|  |  |  | Methanol | NF54 | 67.1 | Inactive | n.d |  |
|  |  |  |  | ENT30 | 97.92 | Inactive | n.d |  |
| *Solanumi incanum* | Solanaceae | Root barks | Water | NF54 | 154.06 | Inactive | n.d |  |
|  |  |  |  | ENT30 | 200 | Inactive | n.d |  |
|  |  |  | Methanol | NF54 | 133.01 | Inactive | n.d |  |
|  |  |  |  | ENT30 | 147.35 | Inactive | n.d |  |
| *Cassia abbreviata* | Leguminosae | Root barks | Water | NF54 | 200 | Inactive | n.d |  |
|  |  |  |  | ENT30 | 200 | Inactive | n.d |  |
|  |  |  | Methanol | NF54 | 200 | Inactive | n.d |  |
|  |  |  |  | ENT30 | 200 | Inactive | n.d |  |
| *Albizia antihelminticas* | Mimosaceae | Root barks | Water | D6 | ˃100 | Inactive | n.d | (Muthaura et al., 2015b) |
|  |  |  |  | W2 | ˃100 | Inactive | n.d |  |
|  |  |  | Methanol | D6 | ˃100 | Inactive | n.d |  |
|  |  |  |  | W2 | ˃100 | Inactive | n.d |  |
| *Albizia antihelminticas* | Leguminosae | Root barks | Water | NF54 | 155.22 | Inactive | n.d | (Rukunga et al., 2009) |
|  |  |  |  | ENT30 | 200 | Inactive | n.d |  |
|  |  |  | Methanol | NF54 | 200 | Inactive | n.d |  |
|  |  |  |  | ENT30 | 200 | Inactive | n.d |  |
| *Albizia amaras* | Leguminosae | Root barks | Water | NF54 | 156.09 | Inactive | n.d |  |
|  |  |  |  | ENT30 | 200 | Inactive | n.d |  |
|  |  |  | Methanol | NF54 | 162.42 | Inactive | n.d |  |
|  |  |  |  | ENT30 | 194.26 | Inactive | n.d |  |
| *Cyperus articulatus* | Cyperaceae | Root barks | Water | NF54 | 7.87 | Good | n.d |  |
|  |  |  |  | ENT30 | 8.59 | Good | n.d |  |
|  |  |  | Methanol | NF54 | 4.84 | Good | n.d |  |
|  |  |  |  | ENT30 | 8.68 | Good | n.d |  |
| *Periploca linearifolia* | Asclepiadaceae | Root barks | Water | NF54 | 134.34 | Inactive | n.d |  |
|  |  |  |  | ENT30 | 152.37 | Inactive | n.d |  |
|  |  |  | Methanol | NF54 | 84.08 | Moderate | n.d |  |
|  |  |  |  | ENT30 | 109.87 | Inactive | n.d |  |
| *Urtica massaica* | Urticacaeae | Roots | Water | NF54 | 189.92 | Inactive | n.d |  |
|  |  |  |  | ENT30 | 196.4 | Inactive | n.d |  |
|  |  |  | Methanol | NF54 | 149.21 | Inactive | n.d |  |
|  |  |  |  | ENT30 | 145.56 | Inactive | n.d |  |
| *Cissampelos pareira* | Menispermaceae | Roots | Water | NF54 | 8.67 | Good | n.d |  |
|  |  |  |  | ENT30 | 8.44 | Good | n.d |  |
|  |  |  | Methanol | NF54 | 5.85 | Good | n.d |  |
|  |  |  |  | ENT30 | 7.7 | Good | n.d |  |
| *Cissampelos pareira* | Menispermaceae | root barrks | Water | D6 | 6.8 | Good | n.d | (Muthaura et al., 2015b) |
|  |  |  |  | W2 | 9.3 | Good | n.d |  |
|  |  |  | Methanol | D6 | 5.2 | Good | n.d |  |
|  |  |  |  | W2 | 6.5 | Good | n.d |  |
| *Maytenus senegalensis* | Celastraceae | Root bark | Water | NF54 | 93.38 | Inactive | n.d | (Rukunga et al., 2009) |
|  |  |  |  | ENT30 | 96.56 | Inactive | n.d |  |
|  |  |  | Methanol | NF54 | 38.24 | Moderate | n.d |  |
|  |  |  |  | ENT30 | 47.78 | Moderate | n.d |  |
| *Erythrina Burtii* | Fabaceae | Root barks | Acetone | D6 | 0.97 | Good | n.d | (Yenesew et al., 2012) |
|  |  |  |  | W2 | 1.73 | Good | n.d |  |
|  |  | stem barks |  | D6 | 2.6 | Good | n.d |  |
|  |  |  |  | W2 | 2.9 | Good | n.d |  |
| *Cyperus articulatus L.* | Cyperaceae | Rhizomes | Methanol | NF54 | 4.8 | Good | n.d | (Rukunga et al., 2008) |
|  |  |  |  | ENT 30 | 8.6 | Good | n.d |  |
|  |  |  | Chloroform | NF54 | 2.1 | Good | n.d |  |
|  |  |  |  | ENT 30 | 3.3 | Good | n.d |  |
|  |  |  | Water | NF54 | ˃50 | Inactive | n.d |  |
|  |  |  |  | ENT 30 | ˃50 | Inactive | n.d |  |
| *Carissa edulis* | Apocynaceae | Roots | Water | ENT 30 | ˃250 | Inactive | brine shrimp | (Kirira et al., 2006) |
|  |  |  |  | NF 54 | 148.53 | Inactive | 260.34 µg/ml |  |
|  |  |  | Methanol | ENT 30 | ˃250 | Inactive |  |  |
|  |  |  |  | NF 54 | ˃250 | Inactive | 186.71µg/ml |  |
| *Neoboutonia macrocalyx* | Euphobiaceae | Stem bark | Water | ENT 30 | 92.85 | Inactive | 41.69µg/ml |  |
|  |  |  |  | NF 54 | 84.56 | Inactive |  |  |
|  |  |  | Methanol | ENT 30 | 78.44 | Inactive | 21.04µg/ml |  |
|  |  |  |  | NF 54 | 78.4 | Inactive |  |  |
| *Acacia nilotica* | Leguminosae | Stem bark | Water | ENT 30 | ˃250 | Inactive | 368.11µg/ml |  |
|  |  |  |  | NF 54 | 153.79 | Inactive |  |  |
|  |  |  | Methanol | ENT 30 | 73.59 | Inactive | 267.31µg/ml |  |
|  |  |  |  | NF 54 | 70.33 | Inactive |  |  |
| *Strychnos heningsii* | Loganiaceae | Stem bark | Water | ENT 30 | 73.39 | Inactive | 293.93µg/ml |  |
|  |  |  |  | NF 54 | 67.16 | Inactive |  |  |
|  |  |  | Methanol | ENT 30 | 190 | Inactive | 101.22µg/ml |  |
|  |  |  |  | NF 54 | 157.91 | Inactive |  |  |
| *Myrica salicifolia* | Myricaeae | Stem bark | Water | ENT 30 | 85.97 | Inactive | 328.22µg/ml |  |
|  |  |  |  | NF 54 | 66.84 | Inactive |  |  |
|  |  |  | Methanol | ENT 30 | 55.89 | Inactive | 320.17µg/ml |  |
|  |  |  |  | NF 54 | 51.01 | Inactive |  |  |
| *Fagaropsis angolensis* | Rutaceae | Stem bark | Water | ENT 30 | 10.65 | Moderate | 173.48µg/ml |  |
|  |  |  |  | NF 54 | 6.13 | Good |  |  |
|  |  |  | Methanol | ENT 30 | 5.04 | Good | 57.09µg/ml |  |
|  |  |  |  | NF 54 | 4.68 | Good |  |  |
| *Zanthoxylum usambarense* | Rutaceae | Stem bark | Water | ENT 30 | 14.33 | Moderate | 260.9µg/ml |  |
|  |  |  |  | NF 54 | 5.25 | Good |  |  |
|  |  |  | Methanol | ENT 30 | 5.54 | Good | 97.66µg/ml |  |
|  |  |  |  | NF 54 | 3.2 | Good |  |  |
| *Harrisonia abyssinica* | Simaroubaceae | Stem bark | Water | ENT 30 | 89.74 | Inactive | 234.71µg/ml |  |
|  |  |  |  | NF 54 | 86.56 | Inactive |  |  |
|  |  |  | Methanol | ENT 30 | 79.5 | Inactive | 217.34µg/ml |  |
|  |  |  |  | NF 54 | 72.66 | Inactive |  |  |
| *Withania somnifera* | Solanaceae | Stem bark | Water | ENT 30 | ˃250 | Inactive | 301.44µg/ml |  |
|  |  |  |  | NF 54 | ˃250 | Inactive |  |  |
|  |  |  | Methanol | ENT 30 | 145.86 | Inactive | 207.27µg/ml |  |
|  |  |  |  | NF 54 | 125.59 | Inactive |  |  |
| *Suregada zanzibariensis* | Euphorbiaceae | Leaves | Water | D6 | >125. | Inactive | HELF Cells >1000 µg/ml | (E. V. Kigondu et al., 2009) |
|  |  |  |  | W2 | >125. | Inactive |  |  |
|  |  |  | Methanol | D6 | 4.66 | Good | >1000µg/ml |  |
|  |  |  |  | W2 | 1.82 | Good |  |  |
| *Aloe nyeriensis var kedongensis* | Aloeaceae | Leaves | Water | D6 | 87.7 | Inactive | >1000µg/ml |  |
|  |  |  |  | W2 | 68.4 | Inactive |  |  |
|  |  |  | Methanol | D6 | >125. | Inactive | >500µg/ml |  |
|  |  |  |  | W2 | >125. | Inactive |  |  |
| *Albizia coriaria* | Fabaceae | Stem bark | Water | D6 | >125 | Inactive | >1000µg/ml |  |
|  |  |  |  | W2 | >125 | Inactive |  |  |
|  |  |  | Methanol | D6 | 37.83 | Moderate | >500µg/ml |  |
|  |  |  |  | W2 | 71.17 | Inactive |  |  |
| *Delonix elata* | Fabaceae | Stem bark | Water | D6 | >125 | Inactive |  |  |
|  |  |  |  | W2 | >125 | Inactive | n.d |  |
|  |  |  | Methanol | D6 | >125 | Inactive |  |  |
|  |  |  |  | W2 | >125 | Inactive |  |  |
| *Acacia tortilis* | Fabaceae | Stem bark | Water | D6 | >125 | Inactive | >1000µg/ml |  |
|  |  |  |  | W2 | >125 | Inactive |  |  |
|  |  |  | Methanol | D6 | >125 | Inactive | >500µg/ml |  |
|  |  |  |  | W2 | 85.73 | Inactive |  |  |
| *Artemisia annua* | Asteraceae | Leaves | Methanol | D6 | 10.4 | Moderate | n.d | (Kangethe et al., 2016) |
| *Baccharoides adoensis* | Asteraceae | Leaves | Pet ether | K1 | ˃30 | Moderate | L6 | (Obbo et al., 2019) |
|  |  |  |  |  |  |  | 4.62µg/ml |  |
|  |  |  | Dichloromethane | K1 | ˃3.3 | Good | 1.04µg/ml |  |
|  |  |  | Methanol | K1 | ˃30 | Moderate | ˃5µg/ml |  |
| *Vernonia amygdalina Delile* | Asteraceae | Leaves | Pet ether | K1 | ˃30 | Moderate | 4.69µg/ml |  |
|  |  |  | Methanol | K1 | ˃30 | Moderate | 2.69µg/ml |  |
| *Schkuhria pinnata (Lam.)* | Asteraceae | Aerial | Hexane | K1 | ˃30 | Moderate | ˃5µg/ml |  |
|  |  |  | Pet ether | K1 | 2.46 | Good | 30µg/ml |  |
| *Momordica foetida Schumach.* | Cucurbitaceae | Roots | Pet ether | K1 | ˃30 | Moderate | 3.48µg/ml |  |
|  |  |  | dichloromethane | K1 | ˃30 | Moderate | 2.62µg/ml |  |
|  |  | Leaves | Pet ether | K1 | ˃30 | Moderate | ˃5µg/ml |  |
| *Entada abyssinica A.Rich.* | Leguminosae | Seeds | Hexane | K1 | ˃30 | Moderate | ˃5µg/ml |  |
|  |  |  | Pet ether | K1 | ˃30 | Moderate | n.d |  |
|  |  |  | Methanol | K1 | ˃10 | Moderate | ˃5µg/ml |  |
| *Entandrophragma utile Sprague.* | Meliaceae | Seeds | Dichloromethane | K1 | ˃30 | Moderate | 4.5µg/ml |  |
|  |  |  | Methanol | K1 | ˃30 | Moderate | ˃5µg/ml |  |
| *Khaya anthotheca (Welw.)* | (Meliaceae) | Seeds | Pet ether | K1 | ˃30 | Moderate | 0.96µg/ml |  |
|  |  |  | Dichloromethane | K1 | ˃30 | Moderate | n.d |  |
|  |  |  | Methanol | K1 | ˃30 | Moderate | n.d |  |
|  |  |  | Water | K1 | ˃30 | Moderate | n.d |  |
| *Agathisanthemum bojeri* | (Rubiaceae) | whole plant | Methanol | D6 | 49.8 | Inactive | n.d | (Muthaura et al., 2015a) |
|  |  |  |  | W2 | 55.9 | Inactive | n.d |  |
| *Acacia nilotica (L.)* | Mimosaceae | stem barks | Water | D6 | ˃100 | Inactive | n.d |  |
|  |  |  |  | W2 | ˃100 | Inactive | n.d |  |
|  |  |  | Methanol | D6 | 70.33 | Inactive | n.d |  |
|  |  |  |  | W2 | 73.6 | Inactive | n.d |  |
| *Acacia seyal Delile* | Mimosaceae | stem barks | Water | W2 | ˃100 | Inactive | n.d |  |
|  |  |  | Methanol | W2 | 89.3 | Inactive | n.d |  |
| *Acalypta fruticosa* | Euphorbiaceae | leaves | Methanol | D6 | 13.8 | Moderate | n.d |  |
| *Adansonia digitata* | Bombacaceae | stem barks | Methanol | D6 | 78.9 | Inactive | n.d |  |
|  |  |  |  | W2 | 67.3 | Inactive | n.d |  |
| *Boscia salicifolia* | Rubiaceae | stem barks | Water | D6 | 3.6 | good | n.d |  |
|  |  |  |  | W2 | 10.1 | Moderate | n.d |  |
|  |  |  | Methanol | D6 | 1.1 | good | n.d |  |
|  |  |  |  | W2 | 8.8 | good | n.d |  |
| *Bridelia micrantha* | Euphorbiaceae | stem barks | Methanol | D6 | 19.4 | Moderate | n.d |  |
|  |  |  |  | W2 | 14.2 | Moderate | n.d |  |
| *Cassia abbreviata* | Fabaceae | root barks | Water | D6 | ˃100 | Inactive | n.d |  |
|  |  |  |  | W2 | ˃100 | Inactive | n.d |  |
|  |  |  | Methanol | D6 | ˃100 | Inactive | n.d |  |
|  |  |  |  | W2 | ˃100 | Inactive | n.d |  |
| *Cassia occidentalis* | Fabaceae | root barks | Methanol | D6 | 18.8 | Moderate | n.d |  |
| *Centella asiatica* | Umbelliferae | whole plant | Water | D6 | 58.6 | Inactive | n.d |  |
|  |  |  | Methanol | W2 | 15.5 | Moderate | n.d |  |
| *Cissampelos mucronata* | Menispermaceae | root barks | Methanol | D6 | 8.8 | good | n.d |  |
|  |  |  |  | W2 | 9.2 | good | n.d |  |
|  |  | leaves | Methanol | D6 | 4.4 | good | n.d |  |
| *Carissa edulis* | Apocynaceae | Root barks | Water | D6 | ˃100 | Inactive | n.d |  |
|  |  |  |  | W2 | ˃100 | Inactive | n.d |  |
|  |  |  | Methanol | D6 | 25.5 | moderate | n.d |  |
| *Commiphora schimperi* | Burseraceae | stem barks | Methanol | D6 | 3.9 | good | n.d |  |
|  |  |  |  | W2 | 5.2 | good | n.d |  |
| *Flacourtia indica* | Flacourtiaceae | leaves | Water | D6 | 89.7 | Inactive | n.d |  |
|  |  |  | Methanol | D6 | 33.7 | Moderate | n.d |  |
| *Acacia senegal* | Mimosaceae | stem bark | Water | W2 | 92.4 | Inactive | n.d | (Muthaura et al., 2015b) |
| *Acacia tortilis* | Fabaceae | stem bark | Water | D6 | ˃100 | Inactive | n.d |  |
|  |  |  | Methanol | D6 | 13.4 | Moderate | n.d |  |
| *Acacia xanthoploea* | Fabaceae | stem barks | Methanol | W2 | 17.3 | Moderate | n.d |  |
| *Ageratum conyzoides* | Asteraceae | whole plant | Methanol | D6 | 11.5 | Moderate | n.d |  |
|  |  |  |  | W2 | 12.1 | Moderate | n.d |  |
| *Ajuga remota* | Labiatae | whole plant | Methanol | D6 | 45.9 | Moderate | n.d |  |
|  |  |  |  | W2 | 77.8 | Inactive | n.d |  |
| *Aloe secundiflora* | Liliaceae | leaves | Water | D6 | 32.61 | Moderate | n.d |  |
|  |  |  | Methanol | D6 | 15.4 | Moderate | n.d |  |
| *Albizia amara* | Mimosaceae | stem barks | Water | D6 | ˃100 | Inactive | n.d |  |
|  |  |  |  | W2 | ˃100 | Inactive | n.d |  |
|  |  |  | Methanol | D6 | ˃100 | Inactive | n.d |  |
|  |  |  |  | W2 | ˃100 | Inactive | n.d |  |
| *Albizia coriaria* | Fabaceae | stem barks | Water | W2 | ˃100 | Inactive | n.d |  |
|  |  |  | Methanol | D6 | 15.2 | Moderate | n.d |  |
|  |  |  |  | W2 | 16.8 | Moderate | n.d |  |
| *Albizia gummifera* | Fabaceae | stem barks | Methanol | W2 | 6.7 | Good | n.d |  |
| *Aspilia pruliseta* | Asteraceae | root barks | Methanol | D6 | 9.7 | Good | n.d |  |
|  |  |  |  | W2 | 6.8 | Good | n.d |  |
| *Aspilia mossambicensis* | Asteraceae | root barks | Methanol | W2 | 44.5 | Moderate | n.d |  |
| *Artemesia afra* | Asteraceae | leaves | Water | D6 | 10.2 | Moderate | n.d |  |
|  |  |  |  | W2 | 4.6 | Good | n.d |  |
|  |  |  | Methanol | D6 | 9.1 | Good | n.d |  |
|  |  |  |  | W2 | 3.9 | Good | n.d |  |
|  |  | stem barks | Water | D6 | 21.6 | Moderate | n.d |  |
|  |  |  |  | W2 | 4.1 | Good | n.d |  |
|  |  |  | Methanol | D6 | 17.8 | Moderate | n.d |  |
|  |  |  |  | W2 | 1.2 | Good | n.d |  |
| *Artemisia annua* | Asteraceae | leaves | Water | D6 | 12.6 | Moderate | n.d |  |
|  |  |  |  | W2 | 14.1 | Moderate | n.d |  |
|  |  |  | Methanol | D6 | 4.7 | Good | n.d |  |
|  |  |  |  | W2 | 5.5 | Good | n.d |  |
| *Balanites aegyptiaca* | Zygophyllaceae | root barks | Methanol | W2 | 8.9 | Good | n.d |  |
| *Berberis holstii* | Berberidaceae | root barks | Methanol | D6 | <5 | Good | n.d |  |
|  |  |  |  | W2 | <5 | Good | n.d |  |
| *Bidens pilosa* | Compositae | leaves | Methanol | D6 | 9.9 | Good | n.d |  |
| *Caesalpinia volkensii* | Caesalpiniaceae | leaves | Methanol | D6 | 33.4 | Moderate | n.d |  |
|  |  |  |  | W2 | 34.2 | Moderate | n.d |  |
| *Catha edulis* | Celastraceae | leaves | Methanol | W2 | <25 | Moderate | n.d |  |
| *Calotropis procera* | Asclepiadaceae | flowers | Methanol | W2 | <25 | Moderate | n.d |  |
| *Cassia didymobotrya F* | Caesalpiniaceae | leaves | Water | D6 | <100 | Inactive | n.d |  |
|  |  |  | Methanol | D6 | 23.4 | Moderate | n.d |  |
| *Clausena anisata* | Rutaceae | stem barks | Water | D6 | 12.6 | Moderate | n.d |  |
|  |  |  |  | W2 | 19.2 | Moderate | n.d |  |
|  |  |  | Methanol | D6 | 8.4 | Good | n.d |  |
|  |  |  |  | W2 | 9.2 | Good | n.d |  |
| *Clematis brachiata* | Ranunculaceae | root barks | Water | D6 | >100 | Inactive | n.d |  |
|  |  |  |  | W2 | 96.2 | Inactive | n.d |  |
| *Clematis hirsuta* | Ranunculaceae | root barks | Methanol | D6 | 58.4 | Inactive | n.d |  |
|  |  |  |  | W2 | 66.5 | Inactive | n.d |  |
| *Clerodendrum johnstonii* | Verbenaceae | root barks | Methanol | D6 | 8.5 | Good | n.d |  |
| *Clerodendrum rotundifolium* | Verbenaceae | root barks | Methanol | D6 | 6.6 | Good | n.d |  |
|  |  |  |  | W2 | 14.5 | Moderate | n.d |  |
|  |  | leaves | DCM | D6 | 3.9 | Good | n.d |  |
|  |  |  |  | W2 | 15.7 | Moderate | n.d |  |
| *Commiphora africana* | Bignoniaceae | stem barks | Methanol | D6 | 10.2 | Moderate | n.d |  |
|  |  |  |  | W2 | 9.6 | Good | n.d |  |
| *Cordia africana* | Boraginaceae | stem bark | Methanol | W2 | 25 | Moderate | n.d |  |
| *Croton macrostachyus* | Euphorbiaceae | stem bark | Methanol | D6 | 3.8 | Good | n.d |  |
|  |  |  |  | W2 | 26.5 | Moderate | n.d |  |
| *Cyperus articulatus* | Cyperaceae | tuber | Water | D6 | 7.9 | Good | n.d |  |
|  |  |  |  | W2 | 8.6 | Good | n.d |  |
|  |  |  | Methanol | D6 | 4.8 | Good | n.d |  |
|  |  |  |  | W2 | 8.7 | Good | n.d |  |
| *Cyathula polycephala* | Amaranthaceae | root barks | Methanol | W2 | 37.2 | Moderate | n.d |  |
| *Cyathula schimperiana* | Amaranthaceae | root barks | Water | D6 | >100 | Inactive | n.d |  |
|  |  |  |  | W2 | >100 | Inactive | n.d |  |
|  |  |  | Methanol | D6 | 5 | Good | n.d |  |
|  |  |  |  | W2 | 17.6 | Moderate | n.d |  |
| *Dichrostachys cineria* | Mimosaceae | stem barks | Water | D6 | >100 | Inactive | n.d |  |
|  |  |  | Methanol | W2 | 59.1 | Inactive | n.d |  |
| *Dodonaea angustifolia* | Sapindaceae | leaves | Water | W2 | >100 | Inactive | n.d |  |
|  |  |  | Methanol | W2 | 32.1 | Moderate | n.d |  |
| *Elaeodendron buchananii* | Celastraceae | stem barks | Water | W2 | >100 | Inactive | n.d |  |
|  |  |  | Methanol | W2 | 17.1 | moderate | n.d |  |
| *Ekerbergia capensis* | Meliaceae | stem barks | Methanol | D6 | 10.5 | moderate | n.d |  |
| *Euclea divinorum* | Ebenaceae | root bark | Methanol | D6 | 6.9 | good | n.d |  |
|  |  |  |  | W2 | 12.4 | moderate | n.d |  |
| *Fagaropsis angolensis* | Rutaceae | stem barks | Water | D6 | 5.5 | good | n.d |  |
|  |  |  |  | W2 | 8.5 | good | n.d |  |
|  |  |  | Methanol | D6 | 4.2 | good | n.d |  |
|  |  |  |  | W2 | 6.9 | good | n.d |  |
| *Ficus sur* | Moraceae | stem bark | Methanol | D6 | 8.5 | good | n.d |  |
|  |  |  |  | W2 | 15.9 | moderate | n.d |  |
|  |  | leaves |  | D6 | 7.9 | good | n.d |  |
|  |  | root barks |  | D6 | 16.5 | moderate | n.d |  |
|  |  |  |  | W2 | 17.3 | moderate | n.d |  |
| *Gomphocarpus semilunatus* | Asclepiadaceae | whole plant | Methanol | W2 | 27.2 | moderate | n.d |  |
| *Hypoestes forskaolii* | Acanthaceae | leaves | Methanol | D6 | 5.9 | good | n.d |  |
|  |  |  |  | W2 | 10.2 | moderate | n.d |  |
|  |  | root barks |  | D6 | 4.3 | good | n.d |  |
|  |  |  |  | W2 | 6.7 | good | n.d |  |
| *Kigelia africana* | Bignoniaceae | leaves | Water | D6 | >100 | Inactive | n.d |  |
|  |  |  |  | W2 | >100 | Inactive | n.d |  |
|  |  |  | Methanol | W2 | 15.9 | moderate | n.d |  |
| *Lantana camara* | Verbenaceae | leaves | Methanol | D6 | <25 | moderate | n.d |  |
| *Lippia javanica* | Verbenaceae | root barks | Methanol | W2 | 5.9 | good | n.d |  |
| *Lonchocarpus eriocalyx* | Fabaceae | stem bark | Water | D6 | >100 | Inactive | n.d |  |
|  |  |  |  | W2 | >100 | Inactive | n.d |  |
|  |  |  | Methanol | D6 | 6.3 | good | n.d |  |
|  |  |  |  | W2 | 35.3 | Moderate | n.d |  |
| *Maytenus heterophylla* | Celastraceae | root bark | Water | D6 | >100 | Inactive | n.d |  |
|  |  |  | Methanol | D6 | 1.8 | good | n.d |  |
|  |  |  |  | W2 | 3.9 | good | n.d |  |
| *Maytenus obtusifolia* | Celastraceae | root bark | Water | D6 | 17.3 | Moderate | n.d |  |
|  |  |  | Methanol | D6 | <1.9 | good | n.d |  |
|  |  |  |  | W2 | 5.8 | good | n.d |  |
| *Melia azedarach* | Meliaceae | stem bark | Methanol | W2 | 100 | Inactive | n.d |  |
| *Microglossa pyrifolia* | Asteraceae | leaves | Methanol | W2 | 10.4 | Moderate | n.d |  |
| *Myrica salicifolia* | Myricaceae | root barks | Water | D6 | 76.8 | Inactive | n.d |  |
|  |  |  |  | W2 | 83.7 | Inactive | n.d |  |
|  |  |  | Methanol | D6 | 51.1 | Inactive | n.d |  |
|  |  |  |  | W2 | 55.9 | Inactive | n.d |  |
| *Neoboutonia macrocalyx* | Eurpobiaceae | stem bark | Water | D6 | 64.8 | Inactive | n.d |  |
|  |  |  |  | W2 | 72.1 | Inactive | n.d |  |
|  |  |  | Methanol | D6 | 44.2 | Moderate | n.d |  |
|  |  |  |  | W2 | 63.4 | Inactive | n.d |  |
| *Olea capensis* | Oleaceae | stem barks | Water | D6 | 81.1 | Inactive | n.d |  |
|  |  |  | Methanol | D6 | 31.9 | Moderate | n.d |  |
| *Olea europaeae* | Oleaceae | stem barks | Water | D6 | 100 | Inactive | n.d |  |
|  |  |  | Methanol | D6 | 17.3 | Moderate | n.d |  |
| *Ocimum americanum* | Labiatae | whole plant | Methanol | D6 | 8.9 | good | n.d |  |
|  |  |  |  | W2 | 12.1 | Moderate | n.d |  |
| *Parinari curatellifolia* | Chrysobalanaceae | root bark | Water | D6 | 21.2 | Moderate | n.d |  |
|  |  |  |  | W2 | 7.9 | good | n.d |  |
|  |  |  | Methanol | D6 | 7.9 | good | n.d |  |
|  |  |  |  | W2 | 3.9 | good | n.d |  |
| *Periploca linearifolia* | Asclepiadaceae | root bark | Water | D6 | >100 | Inactive | n.d |  |
|  |  |  |  | W2 | >100 | Inactive | n.d |  |
|  |  |  | Methanol | D6 | 92.1 | Inactive | n.d |  |
|  |  |  |  | W2 | >100 | Inactive | n.d |  |
| *Plectranthus igniarius* | Labiatae | stem barks | Methanol | W2 | <25 | Moderate | n.d |  |
| *Pittosporum viridiflorum* | Pittosporaceae | leaves | Water | D6 | 27.6 | Moderate | n.d |  |
|  |  |  |  | W2 | >100 | Inactive | n.d |  |
|  |  |  | Methanol | D6 | 18.9 | Moderate | n.d |  |
|  |  |  |  | W2 | 17.6 | Moderate | n.d |  |
| *Prunus africana* | Rosaceae | stem bark | Water | D6 | >100 | Inactive | n.d |  |
|  |  |  | Methanol | D6 | 71.3 | Inactive | n.d |  |
| *Rhamnus prinoides* | Rhamnaceae | root barks | Water | W2 | >100 | Inactive | n.d |  |
|  |  |  | Methanol | W2 | 55.2 | Inactive | n.d |  |
| *Rhamnas staddo* | Rhamnaceae | root barks | Water | W2 | >100 | Inactive | n.d |  |
|  |  |  | Methanol | W2 | 33.4 | Moderate | n.d |  |
| *Rubia cordifolia* | Rubiaceae | whole plant | Methanol | D6 | <5 | good | n.d |  |
|  |  |  |  | W2 | <5 | good | n.d |  |
| *Stephania abbyssinica* | Menispermaceae | root barks | Methanol | D6 | 4.7 | good | n.d |  |
|  |  |  |  | W2 | 6.1 | good | n.d |  |
|  |  | leaves |  | D6 | 4.7 | good | n.d |  |
|  |  |  |  | W2 | 5.2 | good | n.d |  |
| *Strychnos henningsii* | Loganiaceae | stem barks | Water | D6 | >100 | Inactive | n.d |  |
|  |  |  |  | W2 | >100 | Inactive | n.d |  |
|  |  |  | Methanol | D6 | 55.2 | Inactive | n.d |  |
|  |  |  |  | W2 | 66.2 | Inactive | n.d |  |
| *Tarena graveolens* | (Rubiaceae) | stem bark | Methanol | D6 | 33.2 | Moderate | n.d |  |
| *Terminalia brownii* | Combretaceae | fruits | Water | D6 | 41.5 | Moderate | n.d |  |
|  |  |  |  | W2 | 55.8 | Inactive | n.d |  |
|  |  |  | Methanol | D6 | 22.1 | Moderate | n.d |  |
|  |  | stem barks |  | W2 | 33.4 | Moderate | n.d |  |
|  |  |  |  | W2 | 36.6 | Moderate | n.d |  |
| *Teclea simplicifolia* | Rutaceae | stem barks | Water | D6 | 80.4 | Inactive | n.d |  |
|  |  |  |  | W2 | 96.6 | Inactive | n.d |  |
|  |  |  | Methanol | D6 | 67.1 | Inactive | n.d |  |
|  |  |  |  | W2 | 97.9 | Inactive | n.d |  |
| *Tithonia diversifolia* | Asteraceae | leaves | Methanol | W2 | 5.5 | good | n.d |  |
| *Trichilia emetica* | Meliaceae | stem barks | Water | D6 | ˃100 | Inactive | n.d |  |
|  |  |  | Methanol | D6 | 13.3 | Moderate | n.d |  |
|  |  | leaves | Water | D6 | 158 | Inactive | n.d |  |
|  |  |  | Methanol | W2 | 14.4 | Moderate | n.d |  |
| *Trimeria grandifolia* | Gentianaceae | stem barks | Water | D6 | 54.1 | Inactive | n.d |  |
|  |  |  |  | W2 | ˃100 | Inactive | n.d |  |
|  |  |  | Methanol | D6 | 33.7 | Moderate | n.d |  |
|  |  |  |  | W2 | 17.9 | Moderate | n.d |  |
| *Turrea robusta* | Meliaceae | stem barks | Water | D6 | 25.3 | Moderate | n.d |  |
|  |  |  |  | W2 | 42.4 | Moderate | n.d |  |
|  |  |  | Methanol | D6 | 2.1 | good | n.d |  |
|  |  |  |  | W2 | 10.3 | Moderate | n.d |  |
| *Urtica massaica* | Urticaceae | root barks | Water | D6 | ˃100 | Inactive | n.d |  |
|  |  |  |  | W2 | ˃100 | Inactive | n.d |  |
|  |  |  | Methanol | D6 | ˃100 | Inactive | n.d |  |
|  |  |  |  | W2 | ˃100 | Inactive | n.d |  |
|  |  | leaves | Methanol | D6 | ˃100 | Inactive | n.d |  |
|  |  |  |  | W2 | ˃100 | Inactive | n.d |  |
| *Vernonia auriculifera* | Asteraceae | stem barks | Methanol | W2 | 43.5 | Moderate | n.d |  |
| *Vernonia brachycalyx* | Asteraceae | leaves | Methanol | D6 | 27.5 | Moderate | n.d |  |
| *Vernonia lasiopus* | Asteraceae | leaves | Methanol | D6 | 44.3 | Moderate | n.d |  |
|  |  |  |  | W2 | 52.4 | Inactive | n.d |  |
| *Warburgia ugandensis* | Asteraceae | stem barks | Water | D6 | 12.9 | Moderate | n.d |  |
|  |  |  |  | W2 | 15.6 | Moderate | n.d |  |
|  |  |  | Methanol | D6 | 6.4 | good | n.d |  |
|  |  |  |  | W2 | 6.9 | good | n.d |  |
|  |  | root bark | Water | D6 | 6.1 | good | n.d |  |
|  |  |  |  | W2 | 4.1 | good | n.d |  |
|  |  | leaves | Water | D6 | 26.8 | Moderate | n.d |  |
|  |  |  |  | W2 | 33.7 | Moderate | n.d |  |
| *Withania somnifera* | Solanaceae | root barks | Water | D6 | ˃100 | Inactive | n.d |  |
|  |  |  |  | W2 | ˃100 | Inactive | n.d |  |
|  |  |  | Methanol | D6 | ˃100 | Inactive | n.d |  |
|  |  |  |  | W2 | ˃100 | Inactive | n.d |  |
| *Ziziphus abyssinica* | Rhamnaceae | leaves | Methanol | D6 | 17.5 | Moderate | n.d |  |
| *Zanthoxylum usambarense* | Rutaceae | root barks | Water | D6 | 5.2 | good | n.d |  |
|  |  |  |  | W2 | 14.3 | Moderate | n.d |  |
|  |  |  | Methanol | D6 | 3.2 | good | n.d |  |
|  |  |  |  | W2 | 5.5 | good | n.d |  |
| *Azadirachta indica* | (Meliaceae ) | leaves | Water | D6 | 49.5 | Moderate | n.d | (Muthaura et al., 2015a) |
|  |  |  |  | W2 | ˃100 | Inactive | n.d |  |
|  |  |  | Methanol | D6 | 39.5 | Moderate | n.d |  |
|  |  |  |  | W2 | ˃100 | Inactive | n.d |  |
|  |  | stem barks | Water | D6 | 50.1 | Inactive | n.d |  |
| *Chrysanthemum cinerariaefolium* | Asteraceae | Flower | Water | 3D7 | 83.56 | Inactive | VERO cells 83.86 µg/ml | (Wachira et al., 2018) |
|  |  |  |  | W2 | 159.92 | Inactive | 159.92µg/ml |  |
|  |  |  | Methanol | 3D7 | 22 | Moderate | 4.22µg/ml |  |
|  |  |  |  | W2 | 6.55 | Good | 6.55µg/ml |  |
|  |  |  | Pet ether | 3D7 | 16.24 | Moderate |  |  |
|  |  |  |  | W2 | 49.51 | Moderate |  |  |
| *Schkuhria pinnata* | Asteraceae | Whole plant | Water | D6 | 22.5 | Moderate | n.d | (Muthaura et al., 2015b) |
|  |  |  |  | W2 | 51.8 | inactive | n.d |  |
|  |  |  | Methanol | D6 | 1.3 | good | n.d |  |
|  |  |  |  | W2 | 6.8 | good | n.d |  |
| *Clerodendrum eriophyllum* | Verbenaceae | Root barks | Water | K1 | 64 | Inactive |  | (Irungu et al., 2007) |
|  |  |  |  | NF54 | 94.3 | Inactive | 82.6 µg/ml |  |
|  |  |  | Methanol | K1 | 48.2 | Moderate |  |  |
|  |  |  |  | NF54 | 51.5 | Inactive |  |  |
|  |  |  | DCM | K1 | 15.8 | Moderate |  |  |
|  |  |  |  | NF54 | 10.9 | Moderate | 7.9 µg/ml |  |
|  |  | root barks | Water | D6 | 100 | inactive | n.d | (Muthaura et al., 2015b) |
|  |  |  |  | W2 | 100 | inactive | n.d |  |
|  |  |  | Methanol | D6 | 9.5 | good | n.d |  |
|  |  |  |  | W2 | 10.5 | Moderate | n.d |  |
|  |  | Leaves | Methanol | D6 | 1.8 | good | n.d |  |
|  |  |  |  | W2 | 3.9 | good | n.d |  |
| *Clerodendrum eriophyllum* | Verbenaceae | Root bark | Methanol | D6 | 9.51 | Good | 233 µg/ml | (Muthaura et al., 2007) |
|  |  |  |  | W2 | 10.56 | Moderate |  |  |
|  |  |  | Water | D6 | 250 | Inactive | 4571.3 µg/ml |  |
|  |  |  |  | W2 | 250 | Inactive |  |  |
| *Clutia abyssinica* | Euphorbiaceae | Leaves | Methanol | D6 | 7.8 | Good | 331.5 µg/ml |  |
|  |  |  |  | W2 | 11.35 | Moderate |  |  |
|  |  |  | Water | D6 | 65.23 | Inactive | 5574 µg/ml |  |
|  |  |  |  | W2 | 216.81 | Inactive |  |  |
| *Fuerstia africana* | Lamiaceae | Whole plant | Methanol | D6 | 0.98 | Good | 954.7µg/ml |  |
|  |  |  |  | W2 | 2.4 | Good |  |  |
|  |  |  | Water | D6 | 138.26 | Inactive | 4650 µg/ml |  |
|  |  |  |  | W2 | 250 | Inactive |  |  |
| *Schkuhria pinnata* | Asteraceae | whole plant | Methanol | D6 | 1.3 | Good | 161.5 µg/ml |  |
|  |  |  |  | W2 | 6.83 | Good |  |  |
|  |  |  | Water | D6 | 22.51 | Moderate | 3570 µg/ml |  |
|  |  |  |  | W2 | 51.82 | Inactive |  |  |
| *Clutia abyssinica* | Euphorbiaceae | root barks | Methanol | D6 | 9.2 | good | n.d | (Muthaura et al., 2015b) |
|  |  | stem barks | Water | D6 | 98.8 | inactive | n.d |  |
|  |  | Leaves | Water | D6 | 65.2 | inactive | n.d |  |
|  |  |  |  | W2 | 100 | inactive | n.d |  |
|  |  |  | Methanol | D6 | 7.8 | good | n.d |  |
|  |  |  |  | W2 | 11.3 | Moderate | n.d |  |
| *Pittosporum viridiflorum* | Pittosporaceae | Leaves | Methanol | D6 | 18.9 | Moderate | 18.08 µg/ml | (Muthaura et al., 2007) |
|  |  |  |  | W2 | 17.69 | Moderate |  |  |
|  |  |  | Water | D6 | 27.61 | Moderate | 69.21 µg/ml |  |
|  |  |  |  | W2 | 224.27 | Inactive |  |  |
| *Ocotea usambarensis* | Lauraceae | Leaves | Methanol | D6 | 7.69 | Good | 461.5 µg/ml |  |
|  |  |  |  | W2 | 29.75 | Moderate |  |  |
|  |  |  | Water | D6 | 41.17 | Moderate |  |  |
|  |  |  |  | W2 | 76.27 | Inactive |  |  |
| *Vangueria acutiloba* | Rubiaceae | Leaves | Methanol | D6 | 13.36 | Moderate | 661.5 µg/ml |  |
|  |  |  |  | W2 | 33.98 | Moderate |  |  |
|  |  |  | Water | D6 | 178.94 | Inactive |  |  |
|  |  |  |  | W2 | 250 | Inactive |  |  |
| *Boscia angustifolia* | Capparaceae | Leaves | Methanol | D6 | 7.43 | Good | 1000 µg/ml |  |
|  |  |  |  | W2 | 35.93 | Moderate |  |  |
|  |  |  | Water | D6 | 1.42 | Good | 6720 µg/ml |  |
|  |  |  |  | W2 | 4.77 | Good |  |  |
| *Boscia angustifolia* | Capparaceae | stem barks | Water | D6 | 1.4 | good | n.d | (Muthaura et al., 2015b) |
|  |  |  |  | W2 | 4.7 | good | n.d |  |
|  |  |  | Methanol | D6 | 7.4 | good | n.d |  |
|  |  |  |  | W2 | 35.9 | Moderate | n.d |  |
| *Ocotea usambarensis* | Lauraceae | stem barks | Water | D6 | 41.1 | Moderate | n.d |  |
|  |  |  |  | W2 | 76.2 | Inactive | n.d |  |
|  |  |  | Methanol | D6 | 7.6 | good | n.d |  |
|  |  |  |  | W2 | 27.9 | Moderate | n.d |  |
| *Ludwigia erecta* | Onagraceae | Leaves | Methanol | D6 | 4.1 | Good | VERO cells 544.3 µg/ml | (Muthaura et al., 2007) |
|  |  |  |  | W2 | 11.21 | Moderate |  |  |
|  |  |  | Water | D6 | 0.93 | Good | 3283.6 µg/ml |  |
|  |  |  |  | W2 | 1.61 | Good |  |  |
| *Sphaeranthus suaveolens* | Asteraceae | whole plant | Water | D6 | 9.9 | good | n.d | (Muthaura et al., 2015b) |
|  |  |  |  | W2 | 49.2 | Moderate | n.d |  |
|  |  |  | Methanol | D6 | 7.9 | good | n.d |  |
|  |  |  |  | W2 | 56.7 | inactive | n.d |  |
| *Sphaeranthus suaveolens* | Asteraceae | Leaves | Methanol | D6 | 7.93 | Good | 7.93 µg/ml | (Muthaura et al., 2007) |
|  |  |  |  | W2 | 56.73 | Inactive |  |  |
|  |  |  | Water | D6 | 9.98 | Good | 2845.3 µg/ml |  |
|  |  |  |  | W2 | 49.22 | Moderate |  |  |
| *Vangueria acutiloba* | Rubiaceae | stem barks | Water | D6 | ˃100 | inactive | n.d | (Muthaura et al., 2015b) |
|  |  |  |  | W2 | ˃100 | inactive | n.d |  |
|  |  |  | Methanol | D6 | 13.3 | Moderate | n.d |  |
|  |  |  |  | W2 | 33.9 | Moderate | n.d |  |
| *Turrea mombasana* | Meliaceae | whole plant | Methanol | D6 | 6.6 | good | n.d | (Muthaura et al., 2015b) |
|  |  |  | Water | D6 | 39.5 | Moderate | n.d |  |
| *Turraea mombassana* | Meliaceae | Leaves | Water | D6 | 6.1 | Good | ≥100.00 µg/ml | (Nyangacha et al., 2012) |
|  |  |  | Methanol | D6 | 23.92 | Moderate | 22 µg/ml |  |
| *Hugonia castaneifolia Engl.* | Linaceae | Twigs | Water |  |  |  |  |  |
|  |  |  |  | D6 | 8.86 | Good | ≥100.00 µg/ml |  |
|  |  |  | Methanol | D6 | 130.5 | Inactive | ≥100.00 µg/ml |  |
| *Teclea nobilis* | Rutaceae | Stem barks | Water | D6 | ˃100 | inactive | n.d | (Muthaura et al., 2015b) |
|  |  |  | Methanol | D6 | 3.9 | good | n.d |  |
|  |  |  |  | W2 | 29.4 | Moderate | n.d |  |
|  |  | root barks | Methanol | D6 | 4.5 | good | n.d |  |
| *Teclea nobilis Del.* | Rutaceae | Stem bark | Water |  |  |  |  | (Nyangacha et al., 2012) |
|  |  |  |  | D6 | 8.61 | Good | ≥100.00 µg/ml |  |
|  |  |  |  |  |  |  |  |  |
| *Ludwigia erecta* | Onagraceae | whole plant | Water | D6 | 0.9 | Good | n.d | (Muthaura et al., 2015b) |
|  |  |  |  | W2 | 1.6 | Good | n.d |  |
|  |  |  | Methanol | D6 | 4.1 | Good | n.d |  |
|  |  |  |  | W2 | 11.21 | Moderate | n.d |  |
| *Toddalia asiatica (L)* | Rutaceae | Fruits | Ethyl acetate | W2 | 1.87 | Good |  | (Orwa et al., 2013) |
|  |  |  |  |  |  |  |  |  |
|  |  |  |  | D6 | 4.01 | Good |  |  |
|  |  |  | Hexane | W2 | 6.27 | Good |  |  |
|  |  |  |  | D6 | 20.04 | Moderate |  |  |
|  |  |  | Methanol | W2 | 8.24 | Good |  |  |
|  |  |  |  | D6 | 15.13 | Moderate |  |  |
|  |  |  | Water |  |  |  |  |  |
|  |  |  |  | D6 | 10.02 | Moderate |  |  |
|  |  | Root bark | Ethyl acetate | W2 | 5.11 | Good |  |  |
|  |  |  |  | W2 | 7.2 | Good |  |  |
|  |  |  | Hexane | D6 | 5.44 | Good |  |  |
|  |  |  | Methanol | W2 | 2.49 | Good |  |  |
|  |  |  |  | D6 | 16.54 | Moderate |  |  |
|  |  |  | Water | W2 | 2.43 | Good |  |  |
|  |  |  |  |  |  |  |  |  |
|  |  |  |  | D6 | 1.98 | Good |  |  |
|  |  | Leaves | Ethyl acetate | W2 | 6.89 | Good |  |  |
|  |  |  |  | D6 | 2.72 | Good |  |  |
|  |  |  | Hexane | W2 | 12.31 | Moderate |  |  |
|  |  |  |  | D6 | 31.57 | Moderate |  |  |
|  |  |  | Methanol | W2 | 13.35 | Moderate |  |  |
|  |  |  |  | D6 | 21.68 | Moderate |  |  |
|  |  |  | Water | W2 | 8.58 | Good |  |  |
|  |  |  |  |  |  |  |  |  |
|  |  |  |  | W2 | 6.82 | Good |  |  |
|  |  | Root bark | Methanol | D6 | 6.82 | good | n.d | (Muthaura et al., 2015a) |
|  |  |  |  | W2 | 13.9 | Moderate | n.d |  |
| *Fuerstia africana* | Lamiaceae | Whole plant | Water | D6 | >100 | Inactive | n.d | (Muthaura et al., 2015b) |
|  |  |  |  | W2 | >100 | Inactive | n.d |  |
|  |  |  | Methanol | D6 | 0.9 | Good | n.d |  |
|  |  |  |  | W2 | 2.4 | Good |  |  |
| *Pentas lanceolata* | Rubiaceae | Aerial parts | Water | W2 | 43.15 | Moderate |  | (Rotich et al., 2015) |
|  |  |  |  |  |  |  |  |  |
|  |  |  |  | D6 | 3.744 | Good | Vero Cells ≥100µg/ml |  |
|  |  |  | Methanol | W2 | 14.9 | Moderate | ≥100 µg/ml |  |
|  |  |  |  |  |  |  |  |  |
|  |  |  |  | D6 | 17.03 | Moderate |  |  |
| *Fuerstia africana* | Lamiaceae | Aerial parts | Water | W2 | 37.98 | Moderate | ≥100 µg/ml |  |
|  |  |  |  |  |  |  |  |  |
|  |  |  |  | D6 | 1.84 | Good |  |  |
|  |  |  | Methanol | W2 | 6.22 | Good | 63.45 µg/ml |  |
|  |  |  |  |  |  |  |  |  |
|  |  |  |  | D6 | 9.621 | Good |  |  |
| *Ximenia americana* | Olacaceae | Stem barks | Water | W2 | 83.48 | Inactive | 14.3 µg/ml |  |
|  |  |  |  |  |  |  |  |  |
|  |  |  |  | D6 | 2.108 | Good | ≥100 µg/ml |  |
|  |  |  | Methanol | W2 | 36.7 | Moderate |  |  |
|  |  |  |  |  |  |  |  |  |
|  |  |  |  | D6 | 6.45 | Good | ≥100 µg/ml |  |
| *Premna chrysoclada* | Verbenaceae | Stems | Methanol | D6 | 27.63 | Moderate | no | (Gathirwa et al., 2011) |
|  |  |  |  | W2 | 52.35 | Inactive |  |  |
|  |  |  |  | D6 | 0.75 | Good |  |  |
|  |  |  |  | W2 | 9.02 | Moderate |  |  |
|  |  |  |  |  |  |  |  |  |
|  |  | Roots | Methanol | D6 | 27.63 | Moderate | no |  |
|  |  |  |  | W2 | 52.35 | Inactive |  |  |
|  |  | Leaves | Methanol | D6 | 7.75 | Good | no |  |
|  |  |  |  | W2 | 9.02 | Good |  |  |
|  |  |  |  |  |  |  |  |  |
|  |  |  |  | D6 | 11.1 | Moderate |  |  |
| *Uvaria acuminata* | Annonaceae | Leaves | Water | D6 | 51.1 | Inactive | n.d | (Muthaura et al., 2015a) |
|  |  |  |  | W2 | 100 | Inactive | n.d |  |
|  |  | leaves | Methanol | D6 | 8.9 | Good | n.d |  |
|  |  |  |  | W2 | 6.9 | Good | n.d |  |
|  |  | Leaves | Methanol | D6 | 51.13 | Inactive |  | (Gathirwa et al., 2011) |
|  |  |  |  | W2 | 100 | Inactive |  |  |
|  |  | Roots | Methanol | D6 | 8.89 | Good | VERO cells 2.37µg/ml |  |
|  |  |  |  | W2 | 6.9 | Good |  |  |
|  |  |  |  |  |  |  |  |  |
| *Flueggea virosa* | Euphorbiaceae | Leaves | Water | D6 | 25.5 | Moderate | n.d | (Muthaura et al., 2015a) |
|  |  |  |  | W2 | 37.8 | Moderate | n.d |  |
|  |  |  | Methanol | D6 | 2.2 | Good | n.d |  |
|  |  |  |  | W2 | 3.6 | Good | n.d |  |
|  |  | Root barks |  | D6 | 31.5 | Moderate | n.d |  |
|  |  | Roots | Methanol | D6 | 27.05 | Moderate |  | (Gathirwa et al., 2011) |
|  |  |  |  | W2 | 22.34 | Moderate | no |  |
|  |  |  |  |  |  |  |  |  |
|  |  | Aerial parts | Methanol | D6 | 55.03 | Inactive |  |  |
|  |  |  |  | W2 | 55.92 | Inactive |  |  |
| *Azadirachta indica* | Meliaceae | Leaves | Methanol | D6 | 6.24 | Moderate | no |  |
|  |  |  |  | W2 | 7.53 | Moderate |  |  |
|  |  |  |  |  |  |  |  |  |
| *Rhus natalensis* | Anacardiaceae | Roots | Methanol | D6 | 43.92 | Moderate |  |  |
|  |  |  |  | W2 | 51.2 | Inactive | no |  |
|  |  | Leaves | Methanol | D6 | 71 | Inactive |  |  |
|  |  |  |  | W2 | 80.44 | Inactive |  |  |
|  |  |  |  |  |  |  |  |  |
| *Lannea schweinfurthii* | Anacardiaceae | stem barks | Water | D6 | 10.6 | Moderate | n.d | (Muthaura et al., 2015b) |
|  |  |  |  | W2 | 75.8 | inactive | n.d |  |
|  |  |  | Methanol | D6 | 11.4 | Moderate | n.d |  |
|  |  |  |  | W2 | 36.3 | Moderate | n.d |  |
| *Lannea schweinfurthii* | Anacardiaceae | stem barks | Methanol | D6 | 55.96 | inactive | VERO cells 75.8µg/ml | (Gathirwa et al., 2011) |
|  |  |  |  | W2 | 58.54 | inactive |  |  |
|  |  | Leaves |  | D6 | 24.21 | moderate |  |  |
|  |  |  |  | W2 | 33.71 | moderate |  |  |
|  |  |  |  |  |  |  |  |  |
| *Grewia plagiophylla K. Schum* | Tiliaceae | Leaves | Methanol | D6 | 13.28 | Moderate | no |  |
|  |  |  |  | W2 | 34.3 | Moderate |  |  |
|  |  |  |  |  |  |  |  |  |
|  |  | Stem barks | Methanol | D6 | 100 | Inactive |  |  |
|  |  |  |  | W2 | 100 | Inactive |  |  |
| *Hoslundia opposita* | Labietaceae | Roots | Methanol | D6 | 79.38 | Inactive |  |  |
|  |  |  |  | W2 | 64.21 | Inactive | >100 µg/ml |  |
|  |  |  |  |  |  |  |  |  |
|  |  | Aerial parts | Methanol | D6 | 19.73 | Moderate |  |  |
|  |  |  |  | W2 | 29.41 | Moderate |  |  |
|  |  |  |  |  |  |  |  |  |
|  |  | Leaves | Methanol | W2 | 13.22 | Moderate |  |  |
|  |  |  |  |  |  |  |  |  |
| *Combretum padoides* | Combretaceae | Roots | Methanol | D6 | 21.73 | Moderate |  |  |
|  |  |  |  | W2 | 59.43 | Moderate |  |  |
|  |  |  |  |  |  |  |  |  |
| *Combretum illairii* |  | Stem barks | Methanol | D6 | 55.96 | Inactive |  |  |
|  |  |  |  | W2 | 58.54 | Inactive | no |  |
|  |  | leaves |  | D6 | 24.21 | Moderate |  |  |
|  |  |  |  | W2 | 33.71 | Moderate |  |  |
|  |  |  |  |  |  |  |  |  |
| *Combretum padoides* |  |  | Water | D6 | 10.55 | Moderate | n.d | (Muthaura et al., 2015b) |
|  |  |  |  | W2 | 75.9 | Inactive | n.d |  |
|  |  |  |  |  |  |  | n.d |  |
|  |  |  | Methanol | D6 | 11.38 | Moderate | n.d |  |
|  |  |  |  | W2 | 36.26 | Moderate | n.d |  |
|  |  |  |  |  |  |  | n.d |  |
| *Allophylus pervillei* | Sapindaceae | Stem barks | Methanol | D6 | 45.62 | Moderate |  | (Gathirwa et al., 2011) |
|  |  |  |  | W2 | 48.91 | Moderate | no |  |
|  |  |  |  |  |  |  |  |  |
|  |  | Leaves | Methanol | D6 | 100 | Inactive |  |  |
|  |  |  |  | W2 | 100 | Inactive |  |  |
| *Lannea schweinfurthii* | Anacardiaceae | stem barks | Water | D6 | 10.5 | moderate | VERO Cells 3256.52µg/ml | (Gathirwa et al., 2008) |
|  |  |  |  | W2 | 75.9 | inactive |  |  |
|  |  |  |  |  |  |  |  |  |
|  |  |  | Methanol | D6 | 11.38 | moderate | 225.25µg/ml |  |
|  |  |  |  | W2 | 36.26 | Moderate |  |  |
|  |  |  |  |  |  |  |  |  |
| *Sclerocarya birrea* | Anacardiaceae |  | Water | D6 | 18.96 | moderate | 3375.22µg/ml |  |
|  |  |  |  | W2 | 71.74 | inactive |  |  |
|  |  |  |  |  |  |  |  |  |
|  |  |  | Methanol | D6 | 5.91 | good | 361.24µg/ml |  |
|  |  |  |  | W2 | 24.96 | moderate |  |  |
|  |  |  |  |  |  |  |  |  |
| *Turraea robusta* | Meliaceae | Root barks | Water | D6 | 25.32 | Moderate | 45.72µg/ml |  |
|  |  |  |  | W2 | 42.42 | Moderate |  |  |
|  |  |  |  |  |  |  |  |  |
|  |  |  | Methanol | D6 | 2.09 | Good | 24.38µg/ml |  |
|  |  |  |  | W2 | 10.32 | Moderate |  |  |
|  |  |  |  |  |  |  |  |  |
| *Turraea robusta* | Meliaceae | Root barks | Water | K1 | 91.5 | Inactive |  | (Irungu et al., 2007) |
|  |  |  |  | NF54 | 100 | Inactive |  |  |
|  |  |  | Methanol | K1 | 3.5 | Good |  |  |
|  |  |  |  | NF54 | 2.4 | Good |  |  |
| *Sclerocarya birrea,* | Anacardiaceae | Stem barks | Water | D6 | 19.1 | Moderate | n.d | (Muthaura et al., 2015b) |
|  |  |  |  | W2 | 71.7 | inactive | n.d |  |
|  |  |  | Methanol | D6 | 5.9 | Good | n.d |  |
|  |  |  |  | W2 | 24.9 | Moderate | n.d |  |
| *Turraea robusta* | Meliaceae | Stem barks | DCM : Methanol | W2 | 2.87 | Good | VERO cells 21.9 µg/ml | (Irungu et al., 2015) |
|  |  |  |  | D6 | 2.3 | Good | 4TI 5.3µg/ml |  |
|  |  |  |  | W2 | 6.9 | Good | 4TI ND |  |
| *Sclerocarya birrea,* | Anacardiaceae | Root barks | Water | D6 | 10.55 | Moderate |  |  |
|  |  |  |  | W2 | 75.9 | Inactive |  |  |
|  |  |  |  |  |  |  |  |  |
|  |  |  | Methanol | D6 | 11.38 | Moderate |  |  |
|  |  |  |  | W2 | 36.26 | Moderate |  |  |
|  |  |  |  |  |  |  |  |  |
| *Artemisia afra* | Asteraceae | Leaves | Methanol | D6 | 9.04 | Moderate |  | (Gathirwa et al., 2007) |
|  |  |  |  | W2 | 3.98 | Good | Vero cells 594.85µg/ml |  |
|  |  |  |  |  |  |  |  |  |
|  |  |  | Water | D6 | 11.23 | Moderate |  |  |
|  |  |  |  | W2 | 4.65 | Good | 2825.21µg/ml |  |
|  |  |  |  |  |  |  |  |  |
| *Boscia salicifolia* | Capparidaceae | Stem barks | Methanol | D6 | 1.04 | Good |  |  |
|  |  |  |  | W2 | 365 | Inactive | 304.92µg/ml |  |
|  |  |  |  |  |  |  |  |  |
|  |  |  | Water | D6 | 3.65 | Good |  |  |
|  |  |  |  | W2 | 10.09 | Moderate | 1683.95µg/ml |  |
|  |  |  |  |  |  |  |  |  |
| *Catharanthus roseus* | Apocynaceae | Leaves | Methanol | D6 | 4.65 | Good |  |  |
|  |  |  |  | W2 | 5.34 | Moderate | 167.52µg/ml |  |
|  |  |  |  |  |  |  |  |  |
|  |  |  | Water | D6 | 32.36 | Moderate |  |  |
|  |  |  |  | W2 | 36.17 | Moderate | 1285.74µg/ml |  |
|  |  |  |  |  |  |  |  |  |
| *Catharanthus roseus* | Apocynaceae | Leaves | Water | D6 | 32.3 | Moderate | n.d | (Muthaura et al., 2015b) |
|  |  |  |  | W2 | 36.1 | Moderate | n.d |  |
|  |  |  | Methanol | D6 | 4.6 | good | n.d |  |
|  |  |  |  | W2 | 5.3 | good | n.d |  |
| *Rhus natalensis* | Anacardiaceae | fruits | Water | W2 | ˃100 | Inactive | n.d | (Muthaura et al., 2015b) |
|  |  | stem barks |  | D6 | ˃100 | inactive | n.d |  |
|  |  |  |  | W2 | ˃100 | inactive | n.d |  |
|  |  | leaves |  | D6 | 23.9 | Moderate | n.d |  |
|  |  |  |  | W2 | 48.3 | Moderate | n.d |  |
|  |  |  | Methanol | D6 | 9.8 | good | n.d |  |
| *Clutia robusta* | Euphorbiaceae | leaves | Water | D6 | 71.2 | Moderate | n.d | (Muthaura et al., 2015b) |
|  |  |  |  | W2 | 100 | Inactive | n.d |  |
|  |  |  | Methanol | D6 | 3.4 | Good | n.d |  |
|  |  |  |  | W2 | 7.5 | Good | n.d |  |
| *Clutia robusta* | Euphorbiaceae | Leaves | Methanol | D6 | 3.41 | Good |  | (Gathirwa et al., 2007) |
|  |  |  |  | W2 | 7.58 | Moderate | 460.29µg/ml |  |
|  |  |  |  |  |  |  |  |  |
|  |  |  | Water | D6 | 71.24 | Inactive |  |  |
|  |  |  |  | W2 | 157.81 | Inactive | 4352.76µg/ml |  |
|  |  |  |  |  |  |  |  |  |
| *Cyathula schimperiana* | Amaranthaceae | Roots | Methanol | D6 | 10.4 | Moderate |  |  |
|  |  |  |  | W2 | 17.61 | Moderate | 241.34µg/ml |  |
|  |  |  |  |  |  |  |  |  |
|  |  |  | Water | D6 | 175.92 | Inactive |  |  |
|  |  |  |  | W2 | 250 | Inactive | 2907.12µg/ml |  |
|  |  |  |  |  |  |  |  |  |
| *Rhus natalensis* | Anacardiaceae | Stem barks | Methanol | D6 | 76.84 | Inactive |  |  |
|  |  |  |  | W2 | 98 | Inactive | 211.78µg/ml |  |
|  |  |  |  |  |  |  |  |  |
|  |  |  | Water | D6 | 111.6 | Inactive |  |  |
|  |  |  |  | W2 | 105.25 | Inactive | 3858.6µg/ml |  |
|  |  |  |  |  |  |  |  |  |
| *Ximenia americana* | Olacaceae | Root barks | Methanol | D6 | 16.74 | Moderate |  |  |
|  |  |  |  | W2 | 68.19 | Inactive | 198.11µg/ml |  |
|  |  |  |  |  |  |  |  |  |
|  |  |  | Water | D6 | 106.66 | Inactive |  |  |
|  |  |  |  | W2 | 129.23 | Inactive | 1720.38µg/ml |  |
|  |  |  |  |  |  |  |  |  |
| *Ximenia americana* | Olacaceae | Stem barks | Water | D6 | ˃100 | inactive | n.d | (Muthaura et al., 2015b) |
|  |  |  |  | W2 | ˃100 | inactive | n.d |  |
|  |  |  | Methanol | D6 | 18.3 | Moderate | n.d |  |
|  |  |  |  | W2 | 51.3 | inactive | n.d |  |
| *Clerodendrum myricoides* | Verbenaceae | Root barks | Water | K1 | 64 | Inactive |  | (Irungu et al., 2007) |
|  |  |  |  | NF54 | 94.3 | Inactive |  |  |
|  |  |  | Methanol | K1 | 48.2 | Moderate |  |  |
|  |  |  |  | NF54 | 51.5 | Inactive |  |  |
|  |  |  | DCM | K1 | 15.8 | Moderate |  |  |
|  |  |  |  | NF54 | 10.9 | Moderate |  |  |
| *Clerodendrum myricoide* | (Verbenaceae) | root barks | Methanol | D6 | 4.7 | good | n.d | (Muthaura et al., 2015a) |
|  |  |  |  | W2 | 8.3 | good | n.d |  |
|  |  |  | DCM | D6 | 10.2 | Moderate | n.d |  |
|  |  |  |  | W2 | 4.3 | good | n.d |  |
|  |  |  | Methanol | D6 | 18.8 | Moderate | n.d |  |
| *Clerodendrum myricoides* | Verbenaceae | Leaves | Methanol | D6 | 100 | Inactive | n.d | (Jeruto et al., 2015) |
|  |  |  |  | W2 | 20.58 | Moderate | n.d |  |
|  |  |  |  |  |  |  | n.d |  |
|  |  | Root barks | Methanol | D6 | 10.2 | Moderate | n.d |  |
|  |  |  |  | W2 | 9.96 | Moderate | n.d |  |
|  |  |  |  |  |  |  | n.d |  |
| *Asparagus racemosus* | Asparagaceae | Leaves | Chloroform | D6 | 100 | Inactive | n.d |  |
|  |  |  | Methanol | D6 | 31.35 | Moderate | n.d |  |
|  |  |  |  |  |  |  | n.d |  |
|  |  |  | Water | D6 | 125 | Inactive | n.d |  |
|  |  | Whole plant | Methanol | W2 | 42.4 | Moderate | n.d |  |
| *Clutia abbysinica* | Peraceae | Roots | Methanol | D6 | 6.93 | Moderate | n.d | (Muthaura et al., 2015b) |
|  |  |  |  | W2 | 12.22 | Moderate | n.d |  |
|  |  |  |  |  |  | Moderate | n.d |  |
| *Asparagus racemosus* | Liliaceae | Roots | Water | D6 | >125 | Inactive | >1000µg/ml | (E. V. Kigondu et al., 2009) |
|  |  |  |  | W2 | >125 | Inactive |  |  |
|  |  |  | Methanol | D6 | 32.63 | Moderate | >500µg/ml |  |
|  |  |  |  | W2 | 33.95 | Moderate |  |  |
| *Acacia Mellifera* | Leguminosae | Root barks | Methanol | W2 | 8.3 | Good | n.d | (Muthaura et al., 2015b) |
|  |  |  |  | D6 | 10.3 | Moderate | n.d |  |
|  |  |  | DCM | W2 | 4.2 | Good | n.d |  |
|  |  |  |  | D6 | 18.8 | Moderate | n.d |  |
|  |  | Leaves | Methanol | D6 | 3.9 | Good | n.d |  |
|  |  | Stem barks | Methanol | W2 | 5.2 | Good | n.d |  |
|  |  |  |  | D6 | 25.5 | Moderate | n.d |  |
|  |  | Leaves | Water | W2 | 37.8 | Moderate | n.d |  |
|  |  |  | Methanol | D6 | 31.5 | Moderate | n.d |  |
|  |  | Root barks | Methanol | D6 | 89.7 | Inactive | n.d |  |
|  |  | Leaves | Water | D6 | 33.7 | Moderate | n.d |  |
|  |  |  | Methanol | D6 | 18 | Moderate | n.d |  |
| *Sericocomopsis hilde brandtii* | Amaranthacea | Aerial parts | Water | W2 | 78.6 | Inactive | ≥100 | (Rotich et al., 2015) |
|  |  |  |  |  |  |  |  |  |
|  |  |  | Methanol | D6 | 3.15 | Good | ≥100 |  |
|  |  |  |  | W2 | 12.7 | Moderate | ≥100 |  |
|  |  |  |  |  |  |  |  |  |
|  |  |  |  | D6 | 4 | Good | ≥100 |  |
|  |  | Root barks | Water | W2 | 54.2 | Inactive | n.d |  |
|  |  |  |  |  |  |  | n.d |  |
|  |  |  |  | D6 | 2.12 | Good | n.d |  |
|  |  |  | Methanol | W2 | 14.9 | Moderate | n.d |  |
|  |  |  |  |  |  |  | n.d |  |
|  |  |  |  | D6 | 7.127 | Good | n.d |  |
| *Fuerstia africana* | Lamiaceae | Aerial parts | Methanol | W2 | 38.24 | Moderate | n.d | (E. V. Kigondu et al., 2009) |
|  |  |  | Pet ether | D6 | 1.56 | Good | n.d |  |
|  |  |  |  | W2 | 2.5 | Good | n.d |  |
|  |  |  |  | W2 | 14.02 | Moderate | n.d |  |
|  |  | Roots | Methanol | W2 | 28.89 | Moderate | n.d |  |
|  |  |  | Pet ether | D6 | 4.6 | Good | n.d |  |
|  |  |  |  | W2 | 6.3 | Good | n.d |  |
|  |  |  | EtOAc | D6 | 14.1 | Moderate | n.d |  |
|  |  |  |  | W2 | 13.5 | Moderate | n.d |  |
| *Pentas lanceolata* | Rubiaceae | Root barks | Methanol | W2 | 18.2 | Moderate | n.d | (Muthaura et al., 2015b) |
| *Pentas lanceolata* | Rubiaceae | Roots | Methanol | W2 | 50 | Inactive | 366.38µg/ml | (E. V. Kigondu et al., 2009) |
|  |  |  | Pet ether | D6 | 58.5 | Inactive |  |  |
|  |  |  |  | W2 | 100 | Inactive | 23.79 µg/ml |  |
|  |  |  | EtOAc | D6 | 14.3 | Moderate |  |  |
|  |  |  |  | W2 | 37.8 | Moderate | 52.26 µg/ml |  |
|  |  | Aerial parts | Methanol | W2 | 50 | Inactive |  |  |
|  |  |  | Pet ether | W2 | 33.91 | Inactive | >100 µg/ml |  |
|  |  |  | EtOAc | W2 | 37.07 | Moderate |  |  |
| *Fuerstia africana* | Lamiaceae | Whole plant | Water | D6 | >100 | Inactive | n.d | (Muthaura et al., 2015b) |
|  |  |  |  | W2 | >100 | Inactive | n.d |  |
|  |  |  | Methanol | D6 | 0.9 | Good | n.d |  |
|  |  |  |  | W2 | 2.4 | Good | n.d |  |

DCM = Dichloromethane

nd = not done

EtOAc = ethyl acetate

Pet ether = Petroleum ether
