# Supplementary material for: Potential of medicinal plants as antimalarial agents: a review of work done at Kenya Medical Research Institute
Source: Front Pharmacol. 2023 Oct 20;14:1268924. doi: 10.3389/fphar.2023.1268924 (PMC10623325; doi:10.3389/fphar.2023.1268924)
Supplement: Supplementary file 1 [file DataSheet1.ZIP › Table 2.DOCX]

**Supplementary Table S2: Antimalarial activity of plant extracts in mice**

| **Plant screened** | **Family** | **part used** | **Solvent used** | **Parasite Suppression (%)** | **Activity** | **toxicity/LD_50_** | **References** |
| --- | --- | --- | --- | --- | --- | --- | --- |
| *Premna chrysoclada* | Verbenaceae | Stems | methanol | 65.08(250mg/kg) | good | n.d | (Gathirwa et al., 2011) |
|  |  | Leaves | methanol | 65.08(250mg/kg) | good | n.d |  |
| *Uvaria acuminata* | Annonaceae | Roots | methanol | 27(250mg/kg) | inactive | n.d |  |
| *Flueggea virosa* | Euphorbiaceae | Roots | methanol | 68.55(250mg/kg) | good | n.d |  |
| *Azadirachta indica* | Meliaceae | Leaves | methanol | 89.16(250mg/kg) | good | n.d |  |
| *Rhus natalensis* | Anacardiaceae | Leaves | methanol | 82.7(250mg/kg) | good | n.d |  |
| *Lannea schweinfurthii* | Anacardiaceae | Leaves | methanol | 35.72 (250mg/kg) | moderate | n.d |  |
| *Grewia plagiophylla K. Schum* | Tiliaceae | Leaves | methanol | 77.9 (250mg/kg) | good | n.d |  |
| *Hoslundia opposita* | Labietaceae | Roots | methanol | 79.67 (250mg/kg) | good | n.d |  |
|  |  | Aerial parts | methanol | 55.05(250mg/kg) | good | n.d |  |
| *Combretum padoides* | Combretaceae | Roots | methanol | 50.56(250mg/kg | good | n.d |  |
| *Allophylus pervillei* | Sapindaceae | Stem barks | methanol | 62.1(250mg/kg) | good | n.d |  |
| *Lannea schweinfurthii* | Anacardiaceae | stem barks | water | 83.08 (100mg/kg) | good | n.d | (Gathirwa et al., 2008) |
|  |  |  | methanol | 91.37 (100mg/kg) | good | n.d |  |
| *Sclerocarya birrea* | Anacardiaceae | stem barks | water | 66.51 (100mg/kg) | good | n.d |  |
|  |  | stem barks | methanol | 63.49 (100mg/kg) | good | n.d |  |
| *Turraea robusta* | Meliaceae | Root barks | water | 63.8(100mg/kg) | good | n.d |  |
| *Turraea robusta* | Meliaceae | Root barks | methanol | 78.2(100mg/kg) | good | n.d |  |
| *Artemisia afra* | Asteraceae | Leaves | methanol | 77.45(100mg/kg) | good | n.d | (Gathirwa et al., 2007) |
| *Artemisia afra* | Asteraceae | Leaves | water | 70.25(100mg/kg) | good | n.d |  |
| *Boscia salicifolia* | Capparidaceae | Stem barks | methanol | 86.5(100mg/kg) | good | n.d |  |
| *Boscia salicifolia* | Capparidaceae | Stem barks | water | 43.75(100mg/kg) | moderate | n.d |  |
| *Catharanthus roseus* | Apocynaceae | Leaves | methanol | 34.6(100mg/kg) | moderate | n.d |  |
| *Catharanthus roseus* | Apocynaceae | Leaves | water | 42.36(100mg/kg) | moderate | n.d |  |
| *Clutia robusta* | Euphorbiaceae | Leaves | methanol | 23.1(100mg/kg) | inactive | n.d |  |
| *Clutia robusta* | Euphorbiaceae | Leaves | water | 42.35(100mg/kg) | moderate | n.d |  |
| *Cyathula schimperiana* | Amaranthaceae | Roots | methanol | 45.08(100mg/kg) | moderate | n.d |  |
| *Cyathula schimperiana* | Amaranthaceae | Roots | water | 19.06(100mg/kg) | inactive | n.d |  |
| *Rhus natalensis* | Anacardiaceae | Stem barks | methanol | 56.24(100mg/kg) | good | n.d |  |
| *Rhus natalensis* | Anacardiaceae | Stem barks | water | 83.15(100mg/kg) | good | n.d |  |
| *Ximenia americana* | Olacaceae | Root barks | methanol | 36.49(100mg/kg) | moderate | n.d |  |
| *Ximenia americana* | Olacaceae | Root barks | water | 21.55(100mg/kg) | inactive | n.d |  |
| *Clerodendrum myricoides* | Verbenaceae | Leaves | methanol | 82.17(800mg/kg) | good | n.d | (Jeruto et al., 2015) |
| *Clerodendrum myricoides* | Verbenaceae | Root barks | methanol | 61.18(800mg/kg) | good | n.d |  |
| *Asparagus racemosus* | Asparagaceae | Leaves | methanol | 54.35(800mg/kg) | good | n.d |  |
| *Clutia abbysinica* | Peraceae | Roots | methanol | 39.93(800mg/kg) | moderate | n.d |  |
| *Sericocomopsis hilde brandtii* | Amaranthacea | Aerial parts | water | 33.9(500mg/kg) | moderate | n.d | (Rotich et al., 2015) |
| *Sericocomopsis hilde brandtii* | Amaranthacea | Aerial parts | methanol | 17.5(500mg/kg) | inactive | n.d |  |
|  |  | Root barks | water | 30.29(500mg/kg) | moderate | n.d |  |
|  |  | Root barks | methanol | 12.63(500mg/kg) | inactive | n.d |  |
| *Pentas lanceolata* | Rubiaceae | Aerial parts | water | 41(500mg/kg) | moderate | n.d |  |
| *Pentas lanceolata* | Rubiaceae | Aerial parts | methanol | 64.9(500mg/kg) | good | n.d |  |
| *Fuerstia africana* | Lamiaceae | Aerial parts | water | 30.8(500mg/kg) | moderate | n.d |  |
| *Fuerstia africana* | Lamiaceae | Aerial parts | methanol | 27.88(500mg/kg) | inactive | n.d |  |
| *Ximenia americana* | Olacaceae | Stem barks | water | 54.9(500mg/kg) | good | 5000 mg/kg |  |
| *Ximenia americana* | Olacaceae | Stem barks | methanol | 50.8(500mg/kg) | good | n.d |  |
| *Toddalia asiatica (L)* | Rutaceae | Fruits | ethyl acetate | 1.63 | inactive | n.d | (Orwa et al., 2013) |
|  |  |  | water | 17.49 | inactive | n.d |  |
|  |  | root bark | water | 12.15 | inactive | n.d |  |
|  |  | Leaves | water | 23.9 | inactive | n.d |  |
| *Turraea mombassana* | Meliaceae | Leaves | methanol | 52.86(800mg/kg) | good | >5000 mg/kg | (Nyangacha et al., 2012) |
| *Hugonia castaneifolia Engl.* | Linaceae | Twigs | water | 36.6(800mg/kg) | moderate | >5000 mg/kg |  |
|  |  |  | methanol | 46.76(800mg/kg) | moderate | >5000 mg/kg |  |
| *Teclea nobilis Del.* | Rutaceae | Stem bark | water | 33.9(800mg/kg) | moderate | >5000 mg/kg |  |
|  |  |  | methanol | 30.3(800mg/kg) | moderate | >5000 mg/kg |  |
| *Ludwigia erecta* | Onagraceae | Leaves | methanol | 65.28(100mg/kg) | good | >100 mg/kg | (Muthaura et al., 2007) |
| *Ludwigia erecta* | Onagraceae | Leaves | water | 49.64(100mg/kg) | moderate | >5000 mg/kg |  |
| *Sphaeranthus suaveolens* | Asteraceae | Leaves | methanol | 46.74(100mg/kg) | moderate | >100 mg/kg |  |
| *Sphaeranthus suaveolens* | Asteraceae | Leaves | water | 45.38(100mg/kg) | moderate | >5000 mg/kg |  |
| *Vangueria acutiloba* | Rubiaceae | Leaves | methanol | 26.01(100mg/kg) | inactive | >100 mg/kg |  |
| *Vangueria acutiloba* | Rubiaceae | Leaves | water | 39.02(100mg/kg) | moderate | >5000 mg/kg |  |
| *Boscia angustifolia* | Capparaceae | Leaves | methanol | 60.12(100mg/kg) | good | >100 mg/kg |  |
| *Boscia angustifolia* | Capparaceae | Leaves | water | 20.54(100mg/kg) | inactive | >5000 mg/kg |  |
| *Ocotea usambarensis* | Lauraceae | Leaves | methanol | 42.2(100mmg/kg) | moderate | >100 mg/kg |  |
| *Ocotea usambarensis* | Lauraceae | Leaves | water | 31.7(100mg/kg) | moderate | >5000 mg/kg |  |
| *Pittosporum viridiflorum* | Pittosporaceae | Leaves | methanol | 54.77(100mg/kg) | good | 1000 mg/kg |  |
|  |  |  | water | 89.76(100mg/kg) | good | >5000 mg/kg |  |
| *Clutia abyssinica* | Euphorbiaceae | Leaves | methanol | 40.45(100mg/kg) | moderate | >100 mg/kg |  |
|  |  |  | water | 71.69(100mg/kg) | good | >5000 mg/kg |  |
| *Fuerstia africana* | Lamiaceae | Whole plant | methanol | 61.85(100mg/kg) | good | >100 mg/kg |  |
|  |  |  | water | 43.16(100mg/kg) | moderate | >5000 mg/kg |  |
| *Schkuhria pinnata* | Asteraceae | whole plant | methanol | 49.9(100mg/kg) | moderate | >100 mg/kg |  |
|  |  |  | water | 64.22(100mg/kg) | good | >5000 mg/kg |  |
| *Clerodendrum eriophyllum* | Verbenaceae | Root bark | methanol | 90.13(100mg/kg) | good | >100 mg/kg |  |
|  |  |  | water | 61.54(100mg/kg) | good | >5000 mg/kg |  |
| *Chrysanthemum cinerariaefolium* | Asteraceae | Flower | methanol | 10.68(125mg/kg) | inactive | >3000mg/kg | (Wachira et al., 2018) |
|  |  |  |  | 25.22(250mg/kg) | inactive |  |  |
|  |  |  |  | 32.69(500mg/kg) | moderate |  |  |
|  |  |  |  | 45.42(1000mg/kg) | moderate |  |  |
| *Chrysanthemum cinerariaefolium* | Asteraceae | Flower | water | 9.98(125mg/kg) | inactive | >3000 mg/kg |  |
|  |  |  |  | 23.99(250mg/kg) | inactive |  |  |
|  |  |  |  | 29.77(500mg/kg) | inactive |  |  |
|  |  |  |  | 48.04(1000mg/kg) | moderate |  |  |
| *Clausena anisata* | Rutaceae | Stem barks | hexane | 31.4 (100mg/kg) | moderate |  | (Irungu et al., 2012) |
|  |  |  |  | 40.1(250mg/kg) | moderate |  |  |
|  |  |  |  | 56.7(500mg/kg) | good | 4166.7 mg/kg. |  |
|  |  |  | chloroform | 39.8(100mg/kg) | moderate |  |  |
|  |  |  |  | 47.5(250mg/kg) | moderate |  |  |
|  |  |  |  | 73.4(500mg/kg) | good | 4166.7 mg/kg. |  |

**Legend**

Figure in parentheses is the dosage

good (suppression rate of ≥ 50%),

moderate (suppression rate of 30% - 49%) and

inactive (suppression rate of < 30%)(Waiganjo et al., 2020)

Parasite Strain : *Plasmodium berghei* ANKA

Nd = not done
