# Supplementary material for: Potential of medicinal plants as antimalarial agents: a review of work done at Kenya Medical Research Institute
Source: Front Pharmacol. 2023 Oct 20;14:1268924. doi: 10.3389/fphar.2023.1268924 (PMC10623325; doi:10.3389/fphar.2023.1268924)
Supplement: Supplementary file 1 [file DataSheet1.ZIP › Table 3.DOCX]

**Supplementary Table S3: Antiplasmodial activity of isolated compounds**

| **Plant screened** | **Plant Family** | **part used** | **Compound isolated** | **Test** | **Parasite strain** | **IC_50_ ug/ml** | **Activity** | **Cytotoxicity** | **Reference** |
| --- | --- | --- | --- | --- | --- | --- | --- | --- | --- |
| *Turraea nilotica* | Meliaceae | Leaves | Azadirone (**1)** | in vitro | D6 | 23.4 | Moderate | 4TI 14.4 µg/mL | (Irungu et al., 2015) |
|  |  |  |  | In vitro | W2 | 29.6 | Moderate | HEp2 12.8 µg/mL |  |
|  |  |  |  |  |  |  |  | Vero  >229.4 µg/mL |  |
|  |  |  | 12α-Acetoxy-7-deacetylazadirone (**2)** | in vitro | D6 | 31 | Moderate | 4TI 104.6 µg/mL |  |
|  |  |  |  | in vitro | W2 | 30.2 | Moderate | HEp2 4.3 µg/mL |  |
|  |  |  |  |  |  |  |  | Vero  134.9 µg/mL |  |
|  |  |  | Mzikonone (**3**) | in vitro | D6 | 36.6 | Moderate | 4TI 38.8 µg/mL |  |
|  |  |  |  | in vitro | W2 | 40.5 | Moderate | HEp2 59.3 µg/mL |  |
|  |  |  |  |  |  |  |  | Vero  139.6 µg/mL |  |
|  |  |  | 11-epi-Toonacilin (**4)** | in vitro | D6 | 17.4 | Moderate | 4TI 88.6 µg/mL |  |
|  |  |  |  | in vitro | W2 | 14.4 | Moderate | HEp2 68.1 µg/mL |  |
|  |  |  |  |  |  |  |  | Vero  >180.5 µg/mL |  |
|  |  |  | Azadironolide (**5**) | in vitro | D6 | 2.4 | Good | 4TI 14.7 µg/mL |  |
|  |  |  |  | in vitro | W2 | 1.1 | Good | HEp2 8.5 µg/mL |  |
|  |  |  |  |  |  |  |  | Vero  27.6 µg/mL |  |
|  |  |  | Niloticin (**6)** | in vitro | D6 | 48.2 | Moderate | 4TI 14.5 µg/mL |  |
|  |  |  |  | in vitro | W2 | 77 | Inactive | HEp2 6.9 µg/mL |  |
|  |  |  |  |  |  |  |  | Vero  14.5 µg/mL |  |
|  |  |  | Hispidol B (**7)** | in vitro | D2 | 36.8 | Moderate | 4TI 21.7 µg/mL |  |
|  |  |  |  | in vitro | W2 | 37.2 | Moderate | HEp2 7.4 µg/mL |  |
|  |  |  |  |  |  |  |  | Vero  130 µg/mL |  |
|  |  |  | Piscidinol A (**8)** | in vitro | D6 | 37.6 | Moderate | 4TI 8.0 µg/mL |  |
|  |  |  |  | in vitro | W2 | 36.3 | Moderate | HEp2 8.4 µg/mL |  |
|  |  |  |  |  |  |  |  | Vero  41.1 µg/mL |  |
|  |  |  | Niloticin acetate **(9)** | In vitro | D6 | 68.3 | Inactive | 4TI nd |  |
|  |  |  |  | In vitro | W2 | 172.9 | Inactive | HEp2 121.9 µg/mL |  |
|  |  |  |  |  |  |  |  | Vero cells  >200.8 µg/mL |  |
|  |  |  | Piscidinol A diacetate (**10)** | nd | nd | Nd |  | 4TI nd |  |
|  |  |  |  |  |  |  |  | HEp2 15.2 µg/mL |  |
|  |  |  |  |  |  |  |  | Vero cells  >179.2 µg/mL |  |
| *Acacia mellifera* | Leguminosae | Stem barks | Lupeol **(11)** | in vivo | P. Berghei | 16.62 | Inactive |  | (Mutai et al., 2008) |
| *Holarrhena floribunda* | Apocynaceae | Stem bark |  | in vitro | 3D7 | 45 | Moderate |  | (Fotie et al., 2006) |
|  |  |  |  | in vitro | FCR-3 | 41 | Moderate |  |  |
|  |  |  | 3-*O*-(3¢-hydroxyeicosanoyl)lupeol (**12)** | in vitro | 3D7 | 208 | Inactive |  |  |
|  |  |  |  | in vitro | FCR-3 | 198 | Inactive |  |  |
|  |  |  | 3-*O*-[(2¢-(tetracosyloxy)acetyl]lupeol (**13)** | in vitro | 3D7 | 111 | Inactive |  |  |
|  |  |  |  | in vitro | FCR-3 | 69 | Inactive |  |  |
|  |  |  | 3-*O*-[(1¢¢-hydroxyoctadecyloxy)-2¢-hydroxypropanoyl]lupeol (**14**) | in vitro | 3D7 | 391 | Inactive |  |  |
|  |  |  |  | in vitro | FCR-3 | 391 | Inactive |  |  |
| *Ekebergia capensis* | Meliaceae | Roots | 3-oxo-12β-hydroxy-oleanan-28,13β-olide (**15)** | in vitro |  |  |  | HEp2 71.6 µg/ml | (Irungu et al., 2014) |
|  |  |  |  | in vitro |  |  |  | Vero nd |  |
|  |  |  |  | in vitro |  |  |  | 4TI nd |  |
|  |  |  |  | in vitro |  |  |  | HEp2 nd |  |
|  |  |  | oleanonic acid **(16)** | in vitro | D6 | 38.8 | Moderate | MDA-MB-231 nd |  |
|  |  |  |  | in vitro | W2 | 76.7 | inactive | Vero 35.8 µM |  |
|  |  |  |  | in vitro |  |  |  | 4TI 13.3 µM |  |
|  |  |  |  | in vitro |  |  |  | HEp2 1.4 µM |  |
|  |  |  | 3-*epi*-oleanolic acid (**17)** | in vitro | D6 | 205 | Inactive | MDA-MB-231 >212 |  |
|  |  |  |  | in vitro | W2 | 179.4 | Inactive | Vero 58 µM |  |
|  |  |  |  | in vitro |  |  |  | 4TI 30.3 µM |  |
|  |  |  |  | in vitro |  |  |  | HEp2 29.8 µM |  |
|  |  |  | oleanolic acid (**18**) | in vitro | D6 | 49.6 | Moderate | MDA-MB-231 36.54 µM |  |
|  |  |  |  | in vitro | W2 | 82.7 | Inactive | Vero 112 µM |  |
|  |  |  |  | in vitro |  |  |  | 4TI 117.6 µM |  |
|  |  |  |  | in vitro |  |  |  | HEp2 134.9 µM |  |
|  |  |  |  |  |  |  |  | MDA-MB-231 39.8 µM |  |
|  |  |  | ekeberin A (**19**) | in vitro | D6 | 182.2 | Inactive | Vero >219 µM |  |
|  |  |  |  | in vitro | W2 | >219 | Inactive | 4TI 163.2 µM |  |
|  |  |  |  |  |  |  |  | HEp2 > 219 µM |  |
|  |  |  |  |  |  |  |  | MDA-MB-231 nd |  |
|  |  |  | 2-hydroxymethyl-2,3,22,23-tetrahydroxy-6,10,15,19,23-pentamethyl-6,10,14,18-tetracosatetraene (**20)** | in vitro | D6 | 27.1 | Moderate | Vero 35.7 µM |  |
|  |  |  |  | in vitro | W2 | 66.9 | Inactive | 4TI 30.2 µM |  |
|  |  |  |  |  |  |  |  | HEp2 38.4 µM |  |
|  |  |  |  |  |  |  |  | MDA-MB-231 36.69 µM |  |
|  |  |  | 2,3,22,23-tetrahydroxy-2,6,10,15,19,23-hexamethyl-6,10,14,18-tetracosatetraene **(21)** | in vitro | D6 | 56.1 | Inactive | Vero 24.7 µM |  |
|  |  |  |  | In vitro | W2 | 64.3 | Inactive | 4TI 22.5 µM |  |
|  |  |  |  |  |  |  |  | HEp2 35.5 µM |  |
|  |  |  |  |  |  |  |  | MDA-MB->209 |  |
|  |  | Leaves | Proceranolide (**22**) | in vitro | D6 | 84.7 | Inactive | Vero > 213 µM |  |
|  |  |  |  | In vitro | W2 | 150.2 | Inactive | 4TI > 213 µM |  |
|  |  |  |  |  |  |  |  | HEp2 > 213 µM |  |
|  |  |  |  |  |  |  |  | MDA-MB-231 nd |  |
|  |  |  | kaempferol-3-O-β-D-glucopyranoside **(23**) | In vitro | D6 | 97.1 | Inactive | Vero > 213 µM |  |
|  |  |  |  | In vitro | W2 | 105.8 | Inactive | 4TI > 213 µM |  |
|  |  |  |  |  |  |  |  | HEp2 > 213 µM |  |
|  |  |  |  |  |  |  |  | MDA-MB-231 > 213 µM |  |
|  |  |  | quercetin-3-O-β-D-glucopyranoside (**24**) | In vitro | D6 | 42.9 | Moderate | Vero > 216 µM |  |
|  |  |  |  | In vitro | W2 | 105.8 | Inactive | 4TI > 216 µM |  |
|  |  |  |  |  |  |  |  | HEp2 > 216 µM |  |
|  |  |  |  |  |  |  |  | MDA-MB-231 > 216 µM |  |
| *Tephrosia elata* | Leguminosae | Seed pods | Elatadihydrochalcone **(25)** | in vitro | D6 | 2.8 | Good |  | (Muiva et al., 2009) |
|  |  |  |  | in vitro | W2 | 5.5 | Good |  |  |
|  |  |  | β-Acetoxyelatadihydrochalcone (**26**) | in vitro | D6 | 9.6 | Good |  |  |
|  |  |  |  | in vitro | W2 | 12.6 | Moderate |  |  |
|  |  |  | Obovatin (**27**) | in vitro | D6 | 4.9 | Good |  |  |
|  |  |  |  | in vitro | W2 | 6.4 | Good |  |  |
|  |  |  | Obovatin methyl ether (**28**) |  |  |  |  |  |  |
|  |  |  |  | in vitro | D6 | 3.8 | Good |  |  |
|  |  |  |  | in vitro | W2 | 4.4 | Good |  |  |
|  |  |  | Deguelin (**29**) | in vitro | D6 | 6.3 | Good |  |  |
|  |  |  |  | in vitro | W2 | 8.9 | Good |  |  |
| *Tephrosia subtriflora* | Leguminosae | Aerial parts | Subtriflavanonol (**30**) | In vitro | D6 | 12.5 | Moderate | Vero 102.5 µM | (Muiva-Mutisya et al., 2018) |
|  |  |  |  | In vitro | 3D7 | 24.2 | Moderate | HEp 2 16.9 µM |  |
|  |  |  |  | In vitro | KSM | 18.7 | Moderate |  |  |
|  |  |  | MS-II (**31**) | in vitro | D6 | 4.6 | Good | Vero >247.5 µM |  |
|  |  |  |  | in vitro | 3D7 | 1.7 | Good | HEp 2 > 247.5 µM |  |
|  |  |  |  | In vitro | KSM | 1.5 | Good |  |  |
|  |  |  | Spinosaflavanone B (**32)** | in vitro | D6 | 5.9 | Good | nd |  |
|  |  |  |  | in vitro | 3D7 | 5.5 | Good | nd |  |
|  |  |  |  | in vitro | KSM | 22.3 | Moderate |  |  |
|  |  |  | Mundulinol (**33**) | in vitro | D6 | 35.6 | Moderate | Vero cells  >248.7 µM |  |
|  |  |  |  | in vitro | 3D7 | 27.8 | Moderate | HEp 2 cells  >248.7 µM |  |
|  |  |  |  | in vitro | KSM | 22.3 | Moderate |  |  |
| *Drypetes gerrardii* | Rutaceae | Stems | Friedelin **(34)** | in vitro | K1 | 4.8 | Good | L6  > 90 µg/mL | (Ng′ang′a et al., 2012) |
|  |  |  | Epifriedelanol **(35)** | In vitro | K1 | >10 | Moderate | 90 µg/mL |  |
|  |  |  | Friedelanol methyl ether (**36**) | In vitro | K1 | >10 | Moderate | 32.8 µg/mL |  |
|  |  |  | 5 β ,24-cyclofriedelan-3-one (**37)** | In vitro | K1 | 2.2 | Good | 21.2 µg/mL |  |
|  |  |  | 3-epimoretenol (**38)** | In vitro | K1 | >10 | Moderate | 781.9 µg/mL |  |
|  |  |  | Resinone (**39)** | In vitro | K1 | 0.09 | Good | 84.8 µg/mL |  |
|  |  |  | β-sitosterolglucopyranoside (**40**) | In vitro | K1 | 5.4 | good | 14.3 µg/mL |  |
|  |  |  | Amentoflavone **(41)** | In vitro | K1 | 2.6 | good | 0.34 µg/mL |  |
| *Erythrina burtii* | Fabaceae | Stem barks | Burttinol-A (**42)** | in vitro | D6 | 7.6 | good |  | (Yenesew et al., 2012) |
|  |  |  |  | In vitro | W2 | 8.5 | good |  |  |
|  |  |  | Burttinol-B (**43)** | in vitro | D6 | 19.1 | Moderate |  |  |
|  |  |  |  | In vitro | W2 | 21.1 | Moderate |  |  |
|  |  |  | Burttinol-C (**44)** | In vitro | D6 | 9.3 | Good |  |  |
|  |  |  |  | In vitro | W2 | 9.1 | Good |  |  |
|  |  |  | Eryvarin H **(45)** | In vitro | D6 | 13.3 | Moderate |  |  |
|  |  |  |  | In vitro | W2 | 20.3 | Moderate |  |  |
|  |  |  | Burttinol-D **(46)** | In vitro | D6 | 4.9 | Good |  |  |
|  |  |  |  | In vitro | W2 | 6.1 | Good |  |  |
|  |  |  | 4′-O-Methylsigmoidin B **(47)** | In vitro | D6 | 12.4 | Moderate |  |  |
|  |  |  |  | In vitro | W2 | 12.7 | Moderate |  |  |
|  |  |  | Abyssinone V **(48)** | In vitro | D6 | 5.7 | Good |  |  |
|  |  |  |  | In vitro | W2 | 6.6 | Good |  |  |
|  |  |  | Abyssinone V methyl ether (**49)** | In vitro | D6 | 10.7 | Moderate |  |  |
|  |  |  |  | In vitro | W2 | 11.9 | Moderate |  |  |
|  |  |  | Calopocarpin **(50)** | In vitro | D6 | 19.4 | Moderate |  |  |
|  |  |  |  | In vitro | W2 | 17 | Moderate |  |  |
| *Acacia mellifera* | Leguminosae | Stem barks | Acetylated epicatechin(4β-8) catechin; (**51)** | in vivo | P. berghei | 15.67 | Inactive |  | (Mutai et al., 2008) |
|  |  |  | 3-(Z)-trans coumaroylbetulin (**52)** | in vivo | P. berghei | 16.50 | Inactive |  |  |
|  |  |  | 3-(E)-cis coumaroylbetulin (**53**) | in vivo | P. berghei | 16.44 | Inactive |  |  |
|  |  |  | 30-hydroxyl lupan-20 (29)-en -3-on (**54**) | in vivo | P. berghei | 00.82 | Inactive |  |  |
|  |  |  | Betulin (**55**) | in vivo | P. berghei | 24.8 | Inactive |  |  |
